# COVID-19: breaking down a global health crisis

**DOI:** 10.1186/s12941-021-00438-7

**Published:** 2021-05-18

**Authors:** Saad I. Mallah, Omar K. Ghorab, Sabrina Al-Salmi, Omar S. Abdellatif, Tharmegan Tharmaratnam, Mina Amin Iskandar, Jessica Atef Nassef Sefen, Pardeep Sidhu, Bassam Atallah, Rania El-Lababidi, Manaf Al-Qahtani

**Affiliations:** 1 School of Medicine, Royal College of Surgeons in Ireland, Bahrain, Kingdom of Bahrain; 2 The National Taskforce for Combating the Coronavirus (COVID-19), Bahrain, Kingdom of Bahrain; 3 Department of Political Science, Faculty of Arts and Science, University of Toronto, Toronto, Canada; 4 G7 and G20 Research Groups, Munk School of Global Affairs and Public Policy, University of Toronto, Toronto, Canada; 5 School of Medicine, RCSI University of Medicine and Health Sciences, Dublin, Ireland; 6 Department of Pharmacy Services, Cleveland Clinic Abu Dhabi, Al Maryah Island, Abu Dhabi, United Arab Emirates; 7 Cleveland Clinic Lerner College of Medicine of Case Western Reserve University, Cleveland, OH USA; 8 Department of Medicine, Royal College of Surgeons in Ireland, Bahrain, Kingdom of Bahrain; 9 Department of Infectious Diseases, Royal Medical Services, Bahrain Defence Force Hospital, Riffa, Kingdom of Bahrain

**Keywords:** COVID-19, Coronavirus, Pandemic, Global & Public Health, Infectious Diseases

## Abstract

Coronavirus disease 2019 (COVID-19) is the second pandemic of the twenty-first century, with over one-hundred million infections and over two million deaths to date. It is a novel strain from the *Coronaviridae* family, named Severe Acute Respiratory Distress Syndrome Coronavirus-2 (SARS-CoV-2); the 7th known member of the coronavirus family to cause disease in humans, notably following the Middle East Respiratory syndrome (MERS), and Severe Acute Respiratory Distress Syndrome (SARS). The most characteristic feature of this single-stranded RNA molecule includes the spike glycoprotein on its surface. Most patients with COVID-19, of which the elderly and immunocompromised are most at risk, complain of flu-like symptoms, including dry cough and headache. The most common complications include pneumonia, acute respiratory distress syndrome, septic shock, and cardiovascular manifestations. Transmission of SARS-CoV-2 is mainly via respiratory droplets, either directly from the air when an infected patient coughs or sneezes, or in the form of fomites on surfaces. Maintaining hand-hygiene, social distancing, and personal protective equipment (i.e., masks) remain the most effective precautions. Patient management includes supportive care and anticoagulative measures, with a focus on maintaining respiratory function. Therapy with dexamethasone, remdesivir, and tocilizumab appear to be most promising to date, with hydroxychloroquine, lopinavir, ritonavir, and interferons falling out of favour. Additionally, accelerated vaccination efforts have taken place internationally, with several promising vaccinations being mass deployed. In response to the COVID-19 pandemic, countries and stakeholders have taken varying precautions to combat and contain the spread of the virus and dampen its collateral economic damage. This review paper aims to synthesize the impact of the virus on a global, micro to macro scale.

## Introduction

Novel Coronavirus disease 2019 (COVID-19) was crowned as the second pandemic of the twenty-first century by the World Health Organisation (WHO) on March 11th, 2020 [1]. COVID-19 is caused by the Severe Acute Respiratory Distress Syndrome Coronavirus-2 (SARS-CoV-2), a novel strain from the *Coronaviridae* family, first isolated in Wuhan (China) after a cluster of outbreaks. SARS-CoV-2 is a positive-sense, single-stranded enveloped RNA virus that transmits via respiratory droplets and fomites. The virus causes a disease spectrum ranging from asymptomatic to severe acute respiratory distress syndrome (ARDS), and death. Management of this novel disease remains largely supportive, with no approved medications available for treatment [2].

Only after around two months since the initial case report in Wuhan, the first one thousand infections were recorded. Within a short period of time, the infection rate had grown exponentially, and as of February 25, 2021, over one-hundred and thirteen million infections have been registered globally, with over two million deaths (~ 2.2% overall mortality to date, which has been reduced from the ~ 5% mortality at the start of the outbreak) [3, 4]. As global leaders and civil servants worldwide enforce life-altering regulations to contain the disease, scientists scramble to develop timely vaccines, and with healthcare providers treating patients on the frontlines and testing new treatments, it is now more important than ever for the research community to disseminate timely, evidence-based, and up-to-date information about COVID-19 for the public and medical communities alike, both for current and future reference. Therefore, the aim of this review is to provide a holistic, comprehensive overview, both in a retrospective and interim manner, of the relevant epidemiology, pathogenesis, management, potential therapies and vaccines, global efforts, disease burden, and preventive measures that have and can be implemented in the global pursuit of containing COVID-19.

### Search strategy and selection criteria

A range of databases and search strategies were adopted in order to curate a comprehensive overview that addresses the topic from various angles. The WHO Global COVID-19 Database, PubMed, Google Scholar, and medRxiv were mainly searched. Additionally, the WHO and US CDC webpages were often searched for guidelines and data. External webpages such as ClinicalTrials.gov, Our World in Data (OWID), the Oxford COVID-19 Government Response Tracker (OxCGRT), governmental and ministerial webpages, and other UN and Economic related forums and reports were frequently referenced. Alternatives of the ‘COVID-19’ term, such as ‘2019-nCov’, ‘Novel Coronavirus’, ‘Coronavirus 2019’, ‘SARS-CoV-2’, ‘SARS-2’…etc. were used alongside the terms relevant to each sub-header. Non-peer reviewed work was at times referenced due to the exceptional nature of the topic at hand. Papers were only referenced if they were primarily written in English or Arabic, or secondarily referenced in English from a different language. A diverse list of works were reviewed and discussed, ranging from case reports to systematic reviews.

## Epidemiology

**Fig. 1:**
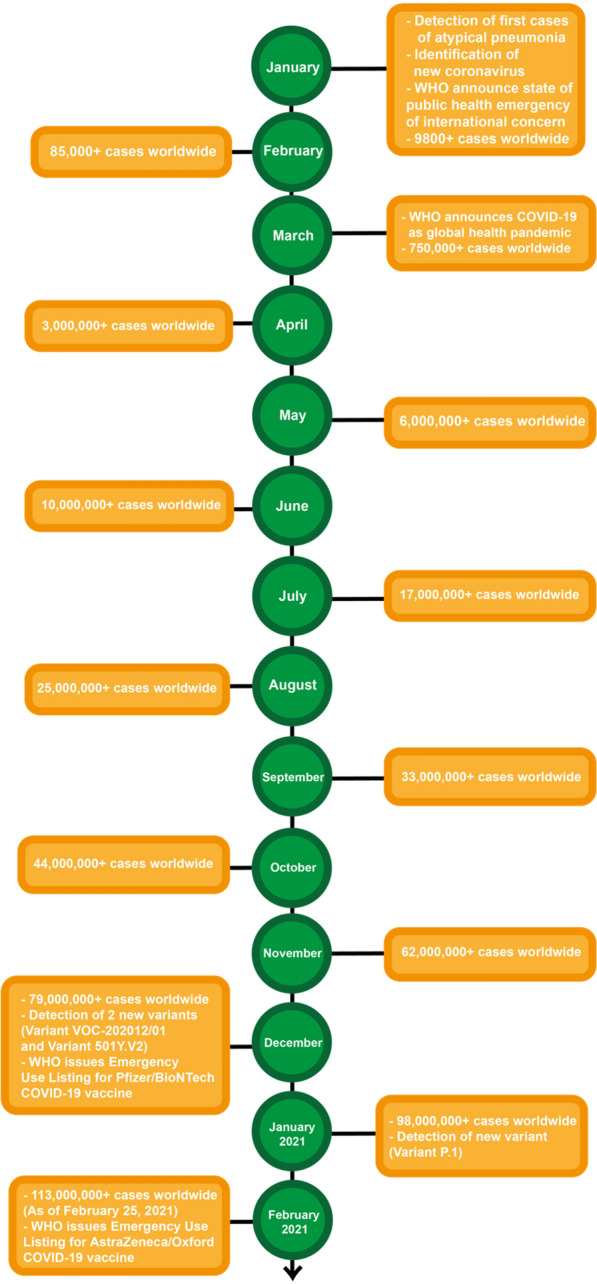
Epidemiologic timeline of events concerning the COVID-19 pandemic [1, 5, 11–13]

In late December 2019, numerous local healthcare institutions in Wuhan, Hubei Province, China had reported several clusters of atypical pneumonia cases (27 cases total) with signs and symptoms greatly resembling those of viral pneumonia, seemingly linked to the South China Seafood City (Huanan Seafood Wholesale Market) [1, 5–7]. Shortly thereafter, on December 31, 2019, the Wuhan Municipal Health Commission issued a notification to the Chinese Center for Disease Control (China CDC). This resulted in a Chinese rapid response team dispatched to undertake immediate investigations, and a subsequent alert issued to the WHO (Fig. 1) [8, 9]. Since then, the Wuhan Seafood Market, which was epidemiologically implicated in the outbreak, was shut down, disinfected, and investigated [5, 7, 9, 10].

In early January 2020, all mimicking etiologies such as the influenza virus, severe acute respiratory syndrome coronavirus (SARS-CoV), and Middle East respiratory syndrome coronavirus (MERS-CoV) were excluded, and the causative agent recognised as a novel coronavirus, now labelled as “severe acute respiratory syndrome coronavirus 2 (SARS-CoV-2)” by the International Committee on Taxonomy of Viruses [7, 9, 14, 15]. It has been genomically sequenced for the first time by scientists of the National Institute of Viral Disease Control and Prevention [16].

The initial transmission event(s), also known as ground zero, are believed to have occurred at the Huanan Seafood Market in Wuhan, via single or multiple animal-to-human transmission events, possibly from bats and pangolins captured and sold at the market. The magnitude of the initial bat-to-human transmission event is not yet known however [7, 16–18]. From those initial cases infected by zoonotic transmission, Chan et al. [19] reported subsequent and successive human-to-human transmission to have occurred. Chan et al. [19], reported a case of a family of six who had travelled to Wuhan from elsewhere in China, with no history of visiting the market, but with a history of visit for only two of the six members to a hospital in Wuhan; the first 2 members contracted the virus from the hospital (possibly from an infected person), and then went on to transmit it to the remaining family members [19]. As such, results of Chan et al. [19] are consistent with person-to-person transmission and with travel-related transmission.

While there has been some speculation regarding alternative origins of the virus, such as it being engineered in a laboratory and subsequently being released or accidentally escaping, Anderson et al., (2020) describes that SARS-CoV-2′s genomic features are highly inconsistent with any laboratory-related scenario of spread/escape, re-emphasising its natural origins with relation to bats [20].

The emergence of SARS-CoV-2 coincided with the Chinese Lunar New Year, which is China’s most celebrated occasion, with millions of people traveling from their residence back to their families and hometowns in other provinces and cities [21]. With an estimated cumulative number of trips amounting to upwards of 3 billion over the 40-day holiday period, an estimated 5 million people had already left Wuhan before the Chinese government implemented a travel ban in late January 2020, making containment of the outbreak difficult [21]. In fact, Zhao et al. [22] found a strong correlation between domestic train-travel from Wuhan to other provinces and the spread of SARS-CoV-2 across China.

**Fig. 2:**
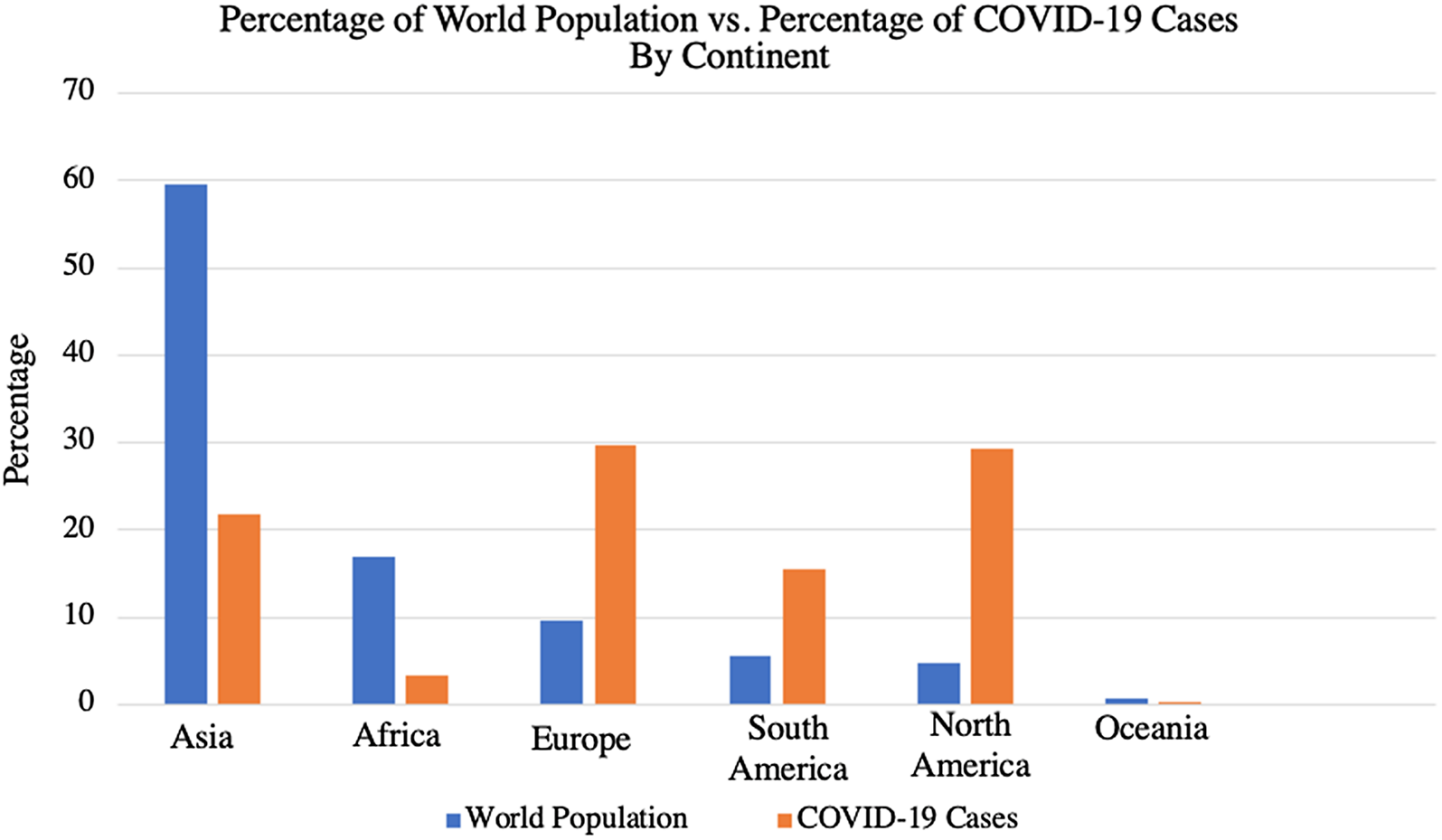
Comparison between prevalence of COVID-19 cases in each continent as a percentage of global cases, and their respective population size as a percentage of global population [11]

Shortly thereafter, positive cases for SARS-CoV-2 began emerging worldwide, facilitated by air travel; both Wuhan and Beijing airports had hundreds of flights to 22 and 54 countries daily, respectively, before the implemented travel bans [23–25]. As of February 25, 2021, the WHO reports a total of 112,224,022 confirmed cases and 2,491,171 confirmed deaths in 236 countries or territories worldwide, equating to a resultant overarching death rate of 2.22% per case of COVID-19 [4]; it is worth mentioning however, that the aforementioned percentage is a simplified calculation based on numbers provided by WHO, and that earlier estimates of the actual global case fatality rate (CFR) vary between 0.3 and 3% [11, 26, 27], with concrete evidence showing CFR to sharply increase with age and comorbidities [28], and by territory [11]. Globally, the list of worst-affected countries includes the United States (28+ million cases; 500,000+ deaths), India (11+ million cases; 156,000+ deaths), Brazil (10+ million cases; 245,000+ deaths), Russia (4+ million cases; 85,000+ deaths), and the United Kingdom [UK] (4+ million cases; 121,000+ deaths). [4]. The spread of COVID-19 has not been proportional to the sizes of the regional populations, which may indicate a range of contributing factors, from containment and screening measures to population demographics (Fig. 2).

During the progression of the outbreak, the situation in Italy had been particularly concerning, with a CFR of 14.44% (95% CI 14.29–14.58) on May 26, 2020 [29]. Additionally, it was reported that Italian infection rates mimic an exponential curve, with unease and doubt regarding whether the Italian healthcare system would be able to cope [30]. Likewise, the outbreak that took place in Iran had been of particular concern, specifically due to the fact that at least six neighbouring countries (Bahrain, Iraq, Kuwait, Oman, Afghanistan, and Pakistan) have reported cases of COVID-19 related to travel from Iran [31].

Likewise, the status of the United States had been just as concerning, being the leading nation in cases and in deaths, housing over a fifth of the total number of infected people worldwide. Even conservative estimates reported in early 2020 showed that the outbreak in the United States may push the American healthcare system beyond its capacity [32]. Indeed The United States had faced a dire shortage in Personal Protective Equipment (PPEs) and ventilators in March 2020 [33], with a small national reserve not equipped for such an unprecedented demand [34]. An increase in ventilator production however soon followed, replenishing the CDC Strategic National Stockpile of Ventilators by September, 2020 [35].

The epicentre of the COVID-19 pandemic has been, and continues to be, dynamic in nature. It had begun in Asia, before transitioning to Europe, then the Americas, and back to Europe (UK) with a variant strain [4, 36]. The WHO had warned that Africa’s increasing infection rates may possibly place it as the next epicentre for the pandemic [37], but that did not seem to manifest.

A number of studies have attempted to model the epidemiological trajectory of the COVID-19 pandemic. Neher et al. (2020), demonstrated that simulation models show a small peak in early 2020, followed by a larger peak in winter 2020/2021 in temperate regions of the Northern Hemisphere [38]. In contrast to this, Wilson et al. (2020) reported that predictions of infection rates and CFRs are highly variable and difficult to establish, given COVID-19’s widespread reach and country-specific infection rates, control efforts, and wildly varying testing and reporting rates [39]. Indeed, an early predictive model set forth by the CoronaTracker Community Research Group anticipated the outbreak to peak before February 20, 2020, which did not occur [40]. The US Centre for Disease Control (CDC) however has been utilising a diverse list of models from various universities and institutes that adopt a range of statistical and machine learning methodologies, to a fair degree of accuracy [41]. Furthermore, attempts at forecasting epidemiologic dynamics via novel markers, such as mean viral loads as indicated by Cycle threshold values, have surfaced [42]. It is worth mentioning, however, that the disease trajectory to date has been exponential: It took around 3 months for the first 500,000 cases to be registered, and a week for the second 500,000. Likewise, it took two weeks to get from the first million to the second, but three days from the 31st to the 32nd million [3]. As of July 2020, countries such as China, Japan, Singapore, and most Middle East countries reported a doubling number of cases between every 5 to 10 days, while the majority of countries, such as the United States, Canada, Italy, Iran, Turkey, and the United Kingdom had cases doubling every 2 to 5 days [43]. This of course was largely fluctuating as the outbreak progressed.

Over time, variant strains of COVID-19 began to appear, often with slightly varying characteristics. The new highly transmissible SARS-CoV-2 strain/variant identified in the UK (London and southeast England) in December, 2020 (named “VUI-202012/01” or “B.1.1.7”) has since spread to many countries, including Ireland, Denmark [44], India [45], and Italy [46]. Since then, other seemingly related and newly identified variants have been implicated in surges of cases in France, South Africa, Israel, Brazil, Japan, and South Korea [44] (REFERENCE 1002), creating public unrest and stress on the already-strained global public health and vaccination efforts to contain COVID-19 [47]. Another novel strain (named “501Y.V2”, which shares one mutation with B.1.1.7 [48], first identified in South Africa [49], has also spread to neighbouring Botswana, as well as distant countries such as the UK and France [44, 48, 49].

## Virulence

The causative agent of COVID-19 is the SARS-CoV-2, which has become the 7th known member of the coronavirus family that causes disease in humans. It is a beta-coronavirus that consists of a long single-stranded positive-sense RNA molecule, surrounded by a lipid envelope that anchors many structural viral glycoproteins, most important of which is the spike glycoprotein [50]. The virus has been found to be about 80% similar in genetic sequence to SARS-CoV, with less similarity to MERS-CoV [18]. An earlier phylogenetic analysis of 103 strains of SARS-CoV-2 in China showed that there are two different types of the virus, an L type and an S type, with the L type forming the majority (70%) of the isolated strains [51].

The SARS-CoV-2 protein likely to be involved in the pathogenesis of COVID-19 is its spike glycoprotein, which has been shown to interact with host cell targets such as the ACE2 receptor and CD26, and is the same viral protein involved in the pathogenesis of SARS [2, 52]. The spike glycoprotein consists of two subunits: S1 (for ACE2 receptor binding), and S2 (for plasma membrane fusion). Upon plasma membrane fusion, the spike protein is cleaved by host proteases, releasing a spike fusion peptide which facilitates viral entry into the host cell [53, 54]. It has been shown that the SARS-CoV-2 spike glycoprotein has a stronger binding affinity to host cell ACE2 receptors than SARS-CoV, and therefore a higher infectious potency [55]. Moreover, the SARS-CoV-2 spike glycoprotein has been shown to contain a unique cleavage site not found in other SARS-like coronaviruses [56]. The identification of the unique features of SARS-CoV-2 such as its spike glycoprotein, the host cell receptors it binds, and the host proteases that act on the virus could be essential in understanding disease pathogenesis, and therefore identifying potential treatment modalities.

The source of SARS-CoV-2 is difficult to confirm, however it most likely originated from bats due to its genetic similarity to bat coronaviruses. Zhou et al. (2020) were the first to display that the SARS-CoV-2 is 96% identical to the bat coronavirus at the whole-genome level [18], and this figure was similarly reported by Yu et al. (2020), who reported that the virus was 96.11% identical to a bat SARS-like coronavirus strain (RaTG13) [57]. It is also yet to be identified whether virus transmission is directly from one organism, or through an intermediate host. Pangolin coronaviruses were found to be 91.02% identical to SARS-CoV-2 at the whole genome level (second most identical after RaTG13 bat coronavirus), and therefore there is great belief that pangolins may be the intermediate hosts for virus transmission to humans [58, 59]. Another study by Zhu et al. (2020) suggested that bats and minks are the two reservoirs of the virus, with minks being the intermediate hosts [60].

## Pathogenesis

The complete pathogenesis of SARS-CoV-2 is yet to be fully comprehended. It is believed that the virus is inhaled through respiratory droplets and acquires entry into the respiratory tract through the nasopharyngeal mucosal membranes. In about 80% of cases, the virus resides in the upper respiratory tract leading to an innate immune response that is mild and requires conservative symptomatic therapy. The remaining 20% of cases experience a much severe form of the disease; the virus diffusely invades and destroys lung alveolar cells, leading to a systemic inflammatory response with ‘cytokine storm’, followed by healing and fibrosis [10, 61]. One study has suggested that intussusceptive angiogenesis may be a part of the pathophysiology of COVID-19; this is a unique disease characteristic when compared to other viral illnesses like influenza. However, this association remains to be further studied and confirmed [62]. Regarding extra-pulmonary manifestations, the virus may disseminate into the blood and affect organs that express ACE2 receptors, such as the lungs, heart, kidneys, and gastrointestinal tract [63, 64].

Disease severity ranges from asymptomatic to severe, with the latter shown to be associated with older age and presence of comorbidities [65]. The most common symptoms being reported are fever and cough [66]. Severe disease involves acute respiratory distress syndrome (ARDS), which can also be associated with severe pneumonia. In fact, the most commonly reported cause of death is respiratory failure [67]. The pneumonia most commonly presents with bilateral multiple lobular and subsegmental areas of ground-glass opacities on CT scan [68]. Non-respiratory complications of COVID-19 may include septic shock (reported in 81.2% of non-survivors in one case series) [69], acute liver injury [70], acute kidney injury [71], ocular problems [72], neurological manifestations [73], and resemblances of disseminated intravascular coagulation (reported in 71% of non-survivors in a case series) [74]. Another complication of increasing concern is the formation of diffuse microvascular thrombi—this has led some health institutions worldwide to recommend thromboprophylaxis for all COVID-19 patients [75].

Several months after the start of the outbreak, a Kawasaki-like disease had been associated with COVID-19 presentation; one province in Italy had detected a 30-fold increase in the incidence of Kawasaki-like disease [76]. Now referred to as multisystem inflammatory syndrome in children (MIS-C), the most common associated signs and symptoms include abdominal pain, vomiting, skin rash, diarrhoea, and hypotension, with a majority having gastrointestinal, cardiovascular, and/or dermatologic or mucocutaneous involvement. The complications are often severe, requiring ICU care in the majority of cases [77].

COVID-19 has also been linked with chemosensory dysfunction; loss of sense of taste and smell has been widely reported in cases of COVID-19, sometimes as the only symptom [78, 79]. This has led the WHO to add loss of smell and/or taste to the official list of COVID-19 symptoms [80].

**Fig. 3:**
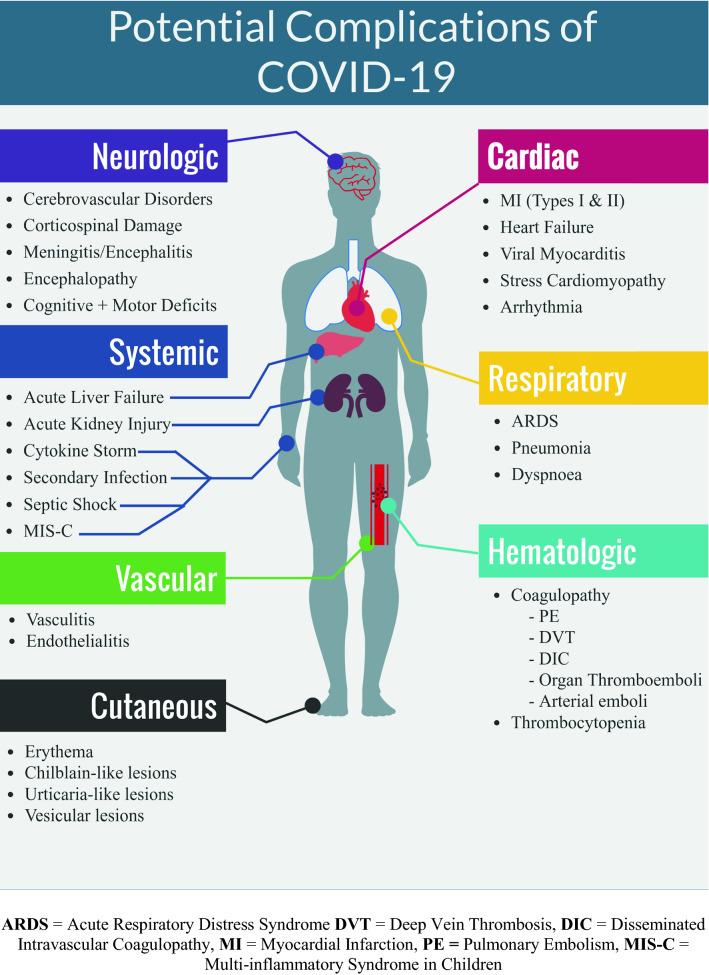
Complications reported to be potentially associated with COVID-19 [77, 82–86]

When compared to SARS and MERS-CoV from a clinical perspective, COVID-19 shares many of the clinical features seen in those diseases; however, it has been associated with fewer occurrences of gastrointestinal and upper respiratory tract symptoms [81]. Several other complications and underlying pathologic mechanisms continue to be reported as potentially associated with COVID-19 (Fig. 3) [77, 82–86].

## Risk factors

The presence of comorbidities has been associated with a worse COVID-19 prognosis; these specifically include cardiovascular disease, diabetes, respiratory disease, and smoking [87, 88]. Elevated levels of blood markers such as lactate dehydrogenase, D-dimers, procalcitonin, serum ferritin, and interleukin-6, as well as leucopoenia, were also found to be associated with worse COVID-19 outcomes, and therefore could potentially be used to monitor disease prognosis [89–91]. The cytokine storm induced by SARS-CoV-2 brings with it a multitude of cytokines; Yang et al. [68] found that interferon gamma induced protein (IP10), interleukin-1 receptor antagonist (IL-1ra), and monocyte chemotactic protein-3 (MCP-3) were significantly associated with increased COVID-19 severity and progression [92]. One case series suggested that thrombocytopenia was significantly associated with death in COVID-19 [93]. The use of non-steroidal anti-inflammatory drugs (NSAIDs) during suspected COVID-19 had also been widely discouraged, due to belief that those drugs may worsen disease outcomes [94]. Recent evidence however seems to suggest there may be no increased risk posed [95]. Other reports hypothesized that ACE inhibitors and nicotine exposure may be associated with cardiorespiratory manifestations in COVID-19 due to upregulation of ACE-2 receptors (which is essential for SARS-COV-2 cell entry), however this remains to be properly studied [96–98]. A protective role however has also been suggested due to the drug limiting angiotensin II related pro-inflammatory signalling, as well as limiting breakdown of bradykinin, which would attenuate hypertension and prevent ventricular apoptosis [99]. In fact, paradoxically, ACE inhibitors suppress TMPRSS2 expression which is an essential co-receptor for SARS-COV-2 cell entry [99]. Evidence on the effect of NSAIDs and ACE inhibitors on COVID-19 outcomes remains inconclusive [100].

## Transmission and precautions

COVID-19 is transmitted from person-to-person through droplet spread, similar to other subtypes of the coronavirus family. The virus may infect a host by coming in contact with any mucosal linings, including mouth, nostrils, and eyes, either directly as respiratory air droplets (suspended in the air when an infected person coughs, sneezes, or talks) or by touching a contaminated surface and then touching a mucosal surface (when droplets rest on a surface; fomites) [101]. COVID-19′s reproduction number (*R*_0_) continues to fluctuate, with estimates from a meta-analysis ranging from 1.4 to 6.49, with a mean of 3.28, a median of 2.79; which is higher than that of SARS [102].

Most worryingly, viral spread can occur through infected asymptomatic individuals, referred to as asymptomatic transmission. This is largely due to the virus’s rather long incubation period, the median of which is estimated around 5.1 (95% CI 4.5 to 5.8) days, but can extend to over 14 days in some cases. By 11.7 days (95% CI 9.7 to 14.2), 95% of people have been shown to demonstrate symptoms of the disease [103].

In a study exploring aerosol and surface stability, SARS-CoV-2 was found to have a very similar profile to SARS-CoV in terms of stability kinetics [104]. The estimated median half-life of SARS-CoV-2 in aerosols is believed to be 1.1 to 1.2 h (95% CI 0.64 to 2.64). Both viruses were more stable on plastic and stainless steel, with viable viruses still detected after 72 h of contamination. The half-life of SARS-CoV-2 on stainless steel and plastic were 5.6 days and 6.8 days respectively. On copper, no viable SARS-CoV-2 was measured after 4 h, in contrast to SARS-CoV-1 which was only undetectable after 8 hours [104]. Contrary to this, on cardboard SARS-CoV-2 lasted longer (24 h) than SARS-CoV-1 (8 h) [104]. The estimated median half-life of SARS-CoV-2 in aerosols is believed to be 1.1 to 1.2 h (95% CI 0.64 to 2.64) [104].

Closed-environments are believed to be grounds for a superspreading event in the transmission of COVID-19. In one study in Japan, 110 positive cases among eleven clusters were contact traced. The study found that the odds of a primary case transmitting COVID-19 in a closed-environment was 18.7 times greater compared to an open-air environment, (95% CI 6.0–57.9). The odds of a superspreading event (defined in this case as transmission to three or more persons), in a closed environment was as high as 29.8 that of an open-air environment (95% CI 5.8–153.4) [105].

The association of weather or meteorological factors with the spread of COVID-19 has been highly contested in public and scientific discourse. One study from China found that meteorological factors play an independent role in COVID-19 transmission. Specifically, that low temperatures, low humidity, and a mild diurnal (daytime) temperature range favours the transmission [106]. On the other hand, another study from China as well, found that after adjustment for relative humidity and ultraviolet (UV) radiation, temperature had no significant association with cumulative incidence rate, indicating that transmission of the virus would not change with increasing temperature. Furthermore, exposure to UV radiation was not significantly associated with cumulative incidence rate after adjusting for temperature and relative humidity either. Relative humidity, maximum temperature, and minimum temperature, were likewise not significantly associated with cumulative incidence rate or the reproduction number of COVID-19 [107].

Although the main mode of SARS-CoV-2 transmission is person-to-person, a number of isolated cases of animals have been reported to test positive for SARS-CoV-2 following close contact with infected humans. Preliminary findings suggest that, of the animal species investigated so far, cats are the most susceptible species to SARS-CoV-2 and can be affected with clinical disease. In the laboratory setting, cats were able to transmit infection to other cats. Ferrets also appear to be susceptible to infection but less so to disease and were also able to transmit infection to other ferrets. Dogs appear to be susceptible to infection but appear to be less affected than ferrets or cats. Egyptian fruit bats were also infected in the laboratory setting but did not show signs of disease or the ability to transmit infection efficiently to other bats. To date, these preliminary findings suggest that poultry and pigs are not susceptible to SARS-CoV-2 infection [108]. Despite this, there is no evidence to suggest that infected animals are playing a role in the spread of COVID-19. Nevertheless, the WHO advises caution at live animal markets and avoiding any direct interaction. Good food safety practices are also recommended especially when dealing with raw animal products [101].

One of the newest COVID variants; B.1.1.7 VOC (Variant of Concern) 202012/01, first detected in the UK and predominantly identified in people younger than 60 years, has been linked to an increasing incidence of COVID-19, but higher mortality or particularly affected groups have not been reported according to the European Centre for Disease Prevention and Control [109]. Upon further investigation, trends have shown that VOC 202012/01 has clear transmission advantage over the non-VOC strain. Epidemiological studies have shown that despite the increased transmissibility, the VOC cases are expected to decline faster than non-VOC cases [110].

An epidemiological study from the Imperial College of London has quantified the transmission advantage of the VOC in comparison to the non-VOC lineages both additively as an increase in R that ranged between 0.4 and 0.7, and also multiplicatively, wherein the increase in R ranged between a 50% and 75% advantage [110]. Additionally, there has been a small shift towards people under their 20 s being more affected by the VOC, but the mechanism that underlies these differences is not yet understood [110].

From a sexual transmission standpoint, SARS-CoV-2 has been detected in the semen of patients with COVID-19 and may also be present in the semen of recovering patients. Owing to the fallibility of the blood-testis/deferens/epididymis barriers, SARS-CoV-2 may be seeded to the male reproductive tract, especially in the presence of systemic local inflammation. It has not been proven, however, that COVID-19 can be spread through sexual transmission [111]. Similarly, the virus has also been detected in other non-respiratory samples such as stool, blood and ocular secretions [112, 113].

With regards to pregnant women, physiological changes in pregnancy and an immunocompromised status could increase susceptibility [114]. However, evidence suggests no risk of increased maternal–fetal transmission. In a cohort of 38 pregnant women, Schwartz et al. (2020) [115] reported no evidence of transplacental or intrauterine transfer. The WHO reports that pregnancy and childbirth do not necessarily aggravate the disease course in the mother [116]. Additionally, the literature is limited with regards to whether COVID-19 can be transmitted in breast milk. In a sample of 6 women, Mullin et al. (2020) [117] were unable to report any findings of the virus in maternal breast milk. However, a symptomatic mother may transmit the illness if in close contact with the neonate. Therefore, social distancing is important, and following appropriate precautions, pumped breast milk may be fed to the neonate by another caregiver. During this process, the mother should ensure she follows strict contact precautions, such as wearing gloves and a face mask, to reduce the risk of transmission, as well as the routine disinfection of surfaces. Note that breastfeeding should not be discouraged unless the mother is acutely ill [118].

The most important public precaution to contain the outbreak remains to be social distancing [119]. It is advised to remain at home except for necessity, which has prompted the implementation of various travel bans, curfews, strict screening procedures, and self-isolation or governmental/hospital quarantine [120]. Moreover, mass gatherings are not advised [121]. The American Academy of Ophthalmology also recommends wearing glasses instead of contact lenses, to decrease eye-touching tendencies [122]. Finally, it is advised to keep a minimum of 2 m distance between individuals to minimise transmission [80].

As of April 3 2020, the CDC also recommends wearing cloth face coverings in public settings, especially in areas of significant community-based transmission [123]. There has been much debate regarding the effectiveness of various forms of face coverings. A meta-analysis of RCTs found that surgical/medical masks offered similar protection against viral respiratory infection (including coronavirus) in health-care workers during non-aerosol generating care as N95 respirator masks [124]. Another systematic review found that that cloth face coverings offered limited efficacy compared to medical grade masks, but can be improved by using a multi-layer cloth mask made of cotton in combination with synthetic cloth material, as well as by improving the fit, and disinfecting it regularly [125]. Finally, a population modelling study found masks of various efficacy to be useful in preventing both illness in health individuals as well as preventing asymptomatic transmission. Hypothetical scenarios of near-universal (80%) adoption of moderately (50%) effective masks in US states were found to potentially prevent 17–45% of projected deaths over two months. Even masks of very low (20%) efficacy were found to be useful if underlying transmission rates were low or decreasing, reducing mortality by up to 24–65% in such scenarios [126]. As such, in view of the evidence, universal face covering/masking has been adopted as a potentially effective public health tool in curtailing community transmission [127].

These extreme measures are necessary to curb the transmission rates and for individuals to protect themselves and others by decreasing the likelihood of exposure to those sick or infected, while also decreasing transmission by infected individuals. The overwhelming of healthcare systems would otherwise be an imminent risk. Other important measures include maintaining hand hygiene and avoiding touching the face after touching other surfaces [128]. Studies on symptomatic patients showed that significant environmental contamination by patients with SARS-CoV-2 through respiratory droplets and fecal shedding suggests that the environment is a potential medium of transmission and supports the need for strict adherence to environmental and hand hygiene and precaution [129].

In addition to the above-mentioned public precautions, the Recommended Interim Infection Prevention and Control (IPC) Recommendations for Patients with Suspected or Confirmed COVID-19 in Healthcare Settings by the CDC provides an extensive list of preventive measures for both healthcare professionals and patients under Section 2 of their guidelines: [https://www.cdc.gov/coronavirus/ 2019-ncov/hcp/infection-control-recommendations.html] [130].

Whether COVID-19 is airborne or not has been a source of uncertainty during the start of the outbreak. In fact, 239 scientists submitted signatories appealing to the medical community and relevant national and international bodies to recognise the potential for airborne spread of COVID-19, via microscopic respiratory droplets at a distance of up to several meters [100]. Experimental data supports the possibility that SARS-COV-2 may be transmitted via aerosols produced emitted during speaking and coughing, which can travel for up to 27 feet. This so-called airborne transmission has become a worry as SARS-COV-2 RNA was shown to be recovered from air-samples in hospitals; underlining the risk of poor ventilation prolonging the amount of time in which aerosols will remain airborne and thus an infection risk [129]. Despite the presence of such data indicating the possibility of aerosol-based transmission, data on infection rates and transmissions in populations during normal daily life has proven difficult to reconcile with long-range airborne/aerosol-based transmission [131].

Nonetheless, for the time being, the use of airborne precautions, specifically the use of the N95 Respirator Masks or equivalent, is warranted in aerosol-producing procedures as declared by the CDC, under Section 1 of their guidelines: [https://www.cdc.gov/coronavirus/2019-ncov/hcp/infection-control-recommendations.html] These procedures include tracheal intubation, non-invasive ventilation, manual ventilation before intubation, bronchoscopy, administration of high-flow oxygen or nebulized medications, tracheotomy, cardiopulmonary resuscitation, and upper endoscopy, but not nasopharyngeal or oropharyngeal specimen collection [132].

Another precautionary method of interest had been the use of hydroxychloroquine in post-exposure prophylaxis. This, however, has not proven to be particularly effective. In a randomized trial of hydroxychloroquine as post-exposure prophylaxis for COVID-19, the incidence of new illness compatible with COVID-19 did not differ significantly between those receiving hydroxychloroquine and participants receiving placebo [133]. Hydroxychloroquine has also completely failed as an effective prophylaxis in a double-blind, placebo-controlled trial among health-care workers [134]. Alternatively, the most promising prophylaxis thus far, is the use of the COVID-19 vaccines [135].

## Screening and diagnosis

Successful containment of COVID-19 is heavily reliant on its accurate diagnosis and efficient population screening. Currently, nucleic acid testing to detect SARS-CoV-2 (RNA genetic identification) is the primary method of diagnosis. Reverse transcription polymerase chain reaction (RT-PCR) kits, using upper or lower respiratory samples, is considered gold standard [136]. Due to shortage of kits, and fairly high false negative rates, CT scans (reported variably with higher sensitivities [137]) have been considered for use in patients with clinical and epidemiologic indications for COVID-19 but a negative RT-PCR [138, 139]. CT scans may also be beneficial as a prognostic test to ascertain disease progression [138, 139]. COVID-19 patients with pneumonia may in fact have lung abnormalities on chest CT (ground-glass opacities), but an initially negative RT-PCR [140]. Of note however, up to approximately 50% of patients with COVID-19 infection may have normal CT scans 0–2 days after onset of flu-like symptoms [138]. Additionally, CT findings, which have much lower specificities thant viral tests, may overlap with many other viral respiratory illnesses and may be completely absent in many positive patients [141]. The former points must thus be considered when diagnosing patients and interpreting the results. The American College of Radiology’s recommendations echo those of the CDC, which emphasise that viral testing (more conventionally, RT-PCR) remains the most specific and confirmatory standard test for COVID-19 [141].

Current progress is being made to develop rapid and accurate point-of-care tests that would reduce the burden on clinical laboratories and speed up the screening process. For instance, the FDA authorized use of a point-of-care test delivering positive results in as little as five minutes and negative results in 13 min. The molecular test identifies a small section of the virus’ genome then amplifies it for detection [142]. Antigen testing, specifically rapid forms, have also been a centre of attention, with some countries making them available commercially [143]. However, antigen tests are generally considered less sensitive than RT-PCR, but just as specific [144, 145]. As such, a negative test should often be followed up by an RT-PCR test, which remains the gold standard for diagnosing COVID-19 [145]. Other tests that are less frequently used or undergoing testing, utilise loop-mediated isothermal amplification, lateral flow, and enzyme-linked immunosorbent assays [100, 107].

As for screening, it is an essential tool for risk communication, and thus outbreak containment. Several studies have attempted to estimate the effectiveness of current common screening procedures. Gostic et al. [146] found that in a growing epidemic, and under best-case assumptions, the median fraction of infected travellers detected is only 0.30 (95% confidence interval: 0.10–0.53). The total fraction detected was found to be lower for programs with only one layer of screening, with arrival screening preferable to departure screening considering possibility of developing symptoms during travel [146]. In a simulation of 100 SARS-CoV-2 infected travellers planning to board a flight, it was estimated for the baseline scenario that 44% (95% CI 33–56) of them would be detected by exit screening, no case (95% CI 0–3) would develop severe symptoms during travel, another 9% (95% CI 2–16) additional cases would be detected by entry screening, and the remaining 46% (95% CI 36–58) would not be detected [147]. Overall, viral testing is only one aspect of what should be a comprehensive approach to outbreak containment via surveillance, including symptom-screening and intensive contact tracing [148].

More innovatively, a study by Qin et al. [149] attempted to create an effective and affordable model to predict new cases in a population. Influenced by the current context of the digital age, social media search indexes (SMSI) for “dry cough”, “fever”, “chest distress”, “coronavirus”, and “pneumonia” were tracked and collected from December 31st, 2019 to February 9th, 2020. SMSI was found to be a predictor of new suspected COVID-19 confirmed cases, and could be detected 6–9 days earlier than the official diagnostic confirmation [149]. This social media-driven approach could therefore be used by national task forces to estimate the new incidence of disease symptoms in the population and prepare accordingly.

## Patient management

Management of COVID-19 is contingent on disease severity. In patients with mild disease, the CDC and WHO recommend home isolation in an effort to alleviate the burden on healthcare systems worldwide. Additionally, in patients with mild disease, hospitalization is not advised unless signs of rapid deterioration are evident, such as respiratory distress [132, 150, 151]. Patients should be educated about important self-isolation measures, such as wearing a face mask at home, disinfecting commonly touched services in co-habited areas, not sharing washrooms or utensils, and practising social distancing. According to the CDC, the decision to discontinue home isolation is contingent on both test and non-test based strategies (see: https://www.cdc.gov/coronavirus/2019-ncov/hcp/disposition-in-home-patients.html). The decision as to which strategy to employ is based on patient and system-level factors such as co-morbidities, immunogenicity, and the availability of testing resources. A non-test-based strategy involves discontinuing home isolation if at least 24 h have passed since resolution of fever without the use of anti-pyretic medications, and other symptoms (e.g. cough, shortness of breath) have improved. In addition, at least ten days must have passed since the appearance of symptom onset. Alternatively, the test-based strategy additionally involves two negative results on nasopharyngeal swabs at least ≥ 24 h apart but is generally not recommended (due to prolonged viral shedding in some cases despite lack of contagiousness) except in cases of severe immunosuppression or if otherwise indicated [152].

In the case of outpatients with mild to moderate disease who are at high risk of disease progression, SARS-CoV-2 neutralizing antibodies (e.g., bamlanivimab plus etesevimab or casirivimab plus imdevimab) may be considered [153, 154].

While patients with mild disease may be able to self-isolate and recover, those with severe disease require hospitalization. This may include complications of SARS-CoV-2 such as pneumonia, ARDS, and sepsis. In response to the COVID outbreak, the CDC has developed a preparedness checklist for hospitals to optimize management of patients from triage to discharge (See: https://www.cdc.gov/coronavirus/2019-ncov/downloads/hcp-preparedness-checklist.pdf], as well as interim-clinical guidance for management of confirmed cases [See: https://www.cdc.gov/coronavirus/2019-ncov/hcp/clinical-guidance-management-patients.html).

Upon admission after triage, regular vital signs should be monitored to prevent clinical deterioration such as septicaemia and ARDS. Antimicrobial agents should also be given if a clinical diagnosis of pneumonia is made. In addition, the use of supplemental oxygen may be warranted with high-flow oxygenation and non-invasive positive pressure ventilation if hypoxemic respiratory failure is suspected. More severe cases may warrant the need for invasive mechanical ventilation or ECMO. Antivirals (e.g. remdesivir) and, more importantly, corticosteroids (e.g. dexamethasone) may be warranted in cases of severe disease. More recently, anti-inflammatories such as tocilizumab have gained interest. The WHO and US National Institute of Health, as well as a wide range of institutions worldwide, continue to update and publish their recommendations as new evidence appears. (A living WHO guideline on drugs for COVID-19: https://www.bmj.com/content/370/bmj.m3379) (The US NIH COVID-19 treatment guidelines: https://www.covid19treatmentguidelines.nih.gov/whats-new/).

Considering the prevalence of coagulopathies as a cause of mortality in COVID-19 patients, standard dose antithrombotic prophylaxis has been recommended in order to circumvent incidences of venous and arterial thrombotic events in hospitalised patients with mild disease. Full-dose therapeutic low-molecular-weight heparin should also be considered in moderately ill hospitalised patients, and should be considered in the case of patients with mild disease who present with indicators of hypercoagulability (e.g. elevated D-Dimer levels) or confirmed VTE (positive point of care ultrasound or CT angiography) [155–159]. Recent data however suggests that in the case of patients with critical illness or those admitted to the ICU, therapeutic dose anticoagulation may worsen mortality-related outcomes due to the increased risk of bleeding—Instead, a prophylactic-intensity anticoagulation dosage is recommended if no thrombosis is suspected or confirmed [160, 161]. A high-intensity pharmacologic thromboprophylaxis (intermediate dose low-molecular-weight heparin) in selected intensive care patients may be ideal to balance between the increased risk of bleeding in this critically ill patient population and the overall COVID-19 related pro-thrombotic state. However, randomized controlled trials are needed to evaluate this strategy. Generally, it is crucial to evaluate overall bleeding and thrombosis tendencies to ensure a personalized management plan informed by the presence of co-morbidities, contraindications (e.g., bleeding tendencies), and other patient-level factors.

### Management in children

The literature suggests that children generally display milder disease and have a better prognosis than adults [162–164]. In a systematic review of 45 studies, Ludvigsson (2020) concluded that children generally have a milder spectrum of disease, and overall, have accounted for only 1–5% of all COVID cases, with death being exceedingly rare [164]. In another study of 2000 children from China, Dong et al. [216] reported that only 13% of children with COVID-19 were symptomatic [165]. However, a limitation of this study was that ‘infected’ status was based on clinical diagnosis and not laboratory confirmation [166]. In another more recent systematic review of clinical manifestations in children with COVID-19, 1124 RT-PCR-confirmed cases from 38 studies were included. Out of the cases with severity classified (n = 1117), 14.2% were asymptomatic, 36.3% mild, 46.0% moderate, 2.1% severe, and 1.2% were critical. It should be noted however that since the results are from patients who presented for medical attention, it is likely that they overestimate the severity of illness in children. Overall, clinicians should have a high level of clinical suspicion, since most cases of COVID-19 in children are asymptomatic or mild, and since reported symptoms of fever and respiratory illness were noted to be not as prevalent as with adult cases [167].

An asymptomatic state could provide the perfect opportunity for children to be implicated in community-based transmission as asymptomatic carriers, and be implicated in family cluster outbreaks, thus emphasising the importance of educating them about maintaining appropriate hygiene, social distancing, and reassurance aimed at mitigating fears regarding the illness. While children may have a better prognosis than adults, this does not necessarily mean they are less susceptible to infection with SARS-CoV-2. In fact, Zheng et al. [162] reported that while children have more favourable prognoses, those < 3 years often had critical illness in the form of pneumonia, which may be due to close contact with a caregiver or family member [162]. Additionally, one retrospective study from.

Pediatric cases in Wuhan suggests that children younger than 2 years were most susceptible to SARS-CoV-2 from the pediatric population [168]. Thus, in hospitalized children management should include intravenous fluids, oxygen support, nutritional aid, and maintaining electrolyte balance [164]. In children with airway compromise, respiratory distress, or suspected sepsis, airway management and oxygen therapy to target SpO2 > 94% are essential to improve clinical outcomes [169]. If mechanical ventilation is unavailable, bubble continuous positive airway pressure (CPAP) is recommended as an alternative [170]. MIS-C has additionally been a major concern in the pediatric population. Management is often supportive, but may include anti-inflammatory measures (e.g., administration of intravenous Immunoglobulins and steroids). Aspirin for concerns regarding coronary artery involvement as well as thrombotic prophylaxis due to associated hypercoagulable state, may also be considered [171]. See: (https://www.who.int/publications-detail/clinical-management-of-severe-acute-respiratory-infection-when-novel-coronavirus-(ncov)-infection-is-suspected) for complete management of the hospitalized pediatric patients.

### Management in pregnancy

Currently, a paucity of data exists on COVID-19 and management during pregnancy. The American College of Obstetricians and Gynecologists (ACOG) recommends that management in pregnant individuals should be the same as non-pregnant females (see: https://www.acog.org/-/media/project/acog/acogorg/files/pdfs/clinical-guidance/practice-advisory/covid-19-algorithm.pdf). However, a review of guidelines recommends designating an area for COVID-19 positive pregnant patients, or those under investigation. Additionally, early discharge from hospital (one day for vaginal delivery and two days for cesarean delivery) is encouraged to reduce risk of transmission [118]. However, despite the evidence, there remains limited literature on the effects of SARS-CoV-2 on pregnant females, such as its effects on the fetus and on labour, if any; more robust studies are thus warranted.

## Cardiovascular controversies

Cardiovascular disease and injury has been reported as both a co-morbidity associated with severe disease, and a complication associated with mortality [87, 172]. SARS-CoV-2’s cellular entry via ACE2 receptors has implicated the heart, where these receptors have been reported to be present [173]. This fact triggers multiple controversies regarding the interplay of the virus with the cardiovascular system. The first question of whether patients with chronically up-regulated ACE2 receptors, such as those on ACE inhibitors, are more prone to viral uptake has been a topic of debate. The second being the need to stop, start, or continue such medications and their effect on the progression of the virus. The ACE-like enzyme appears to partially reverse the effects of its homolog by reverse converting angiotensin II to angiotensin 1–7. This will theoretically result in lessening the known vasoconstriction and remodelling effects associated with the renin–angiotensin–aldosterone system (RAAS), which is a hypothesis that has been utilized to explain the benefits of this strategy in animal models [174, 175]. The lack of data and randomized trials on humans have led many prominent cardiovascular societies to advise against changing clinical practice with regard to the use of RAAS inhibition for the sole purpose of mitigating the pandemic, and instead to continue the standard indication-based utilization. In fact, a case-population study has demonstrated no increase in risk, and even a decreased COVID-19 risk associated with use of RAAS inhibitors in certain populations [176]. However, the use of RAAS inhibition in general is avoided in the setting of vasoplegic shock, which continues to apply for those COVID-19 patients who progress to what has been recently described as stage III (severe) or systemic hyperinflammation [89].

The other main controversy that stemmed from the ACE2 receptor binding mechanism is that of cardiac injury observed in COVID-19, particularly in those that progress to severe disease. Epidemiologic data from Shi et al. (2020) has not only highlighted the common occurrence of such injury but also proved its association with higher mortality through regression models [172, 177]. What continues to be debated is the etiology of said cardiac injury; the first theory being inflammatory cytokine storm-mediated injury rather than an isolated myocardial injury that may be associated with an imbalance in oxygen supply and demand. The other perspective is a direct viral injury caused by the viral binding to the ACE2 cardiac receptors (leading to myocarditis). In either case, it seems reasonable to monitor cardiac troponins, particularly high sensitivity troponin at baseline and then at set intervals when elevated in all hospitalized COVID-19 patients [178]. This is relevant due to the aforementioned association of cardiac injury with mortality, as well as given the results of a recent meta-analysis of all COVID-19 studies that included troponin measurements, highlighting the specific elevation in those with severe infection [179].

Several of the potential medications in the treatment of COVID-19 have QT prolonging potential including lopinavir/ritonavir, azithromycin, and both chloroquine and hydroxychloroquine [180], and thus risk of torsades de pointes (TdP) and sudden cardiac death. Lack of clinical data with favipiravir also suggests the need for monitoring. QT prolongation after single oral doses of favipiravir 1200 mg and 2400 mg has not been reported with this agent except in one case report, where it was found to prolong the QT at higher doses [181, 182].

Below is a suggested protocol for monitoring patients on agents with QT prolonging potential:

1. Discontinue and avoid all other non-critical QT prolong agents
2. Assess baseline ECG, renal and hepatic function, serum potassium and magnesium
3. When possible, have an experienced cardiologist/electrophysiologist measure QTc, and seek pharmacist input in the setting of acute renal or hepatic failure
4. Assess baseline risk of QT prolongation using the Tisdale risk score [183]
5. Relative contraindications: history of long QT syndrome or baseline QTc > 500ms
6. Ongoing monitoring includes telemetry, laboratory studies, and ECG 2–3 h after the second dose and daily thereafter
7. Duration of use of these medications for COVID-19 infection is short (5 to 10 days for acute illness)

## Vaccination efforts

The ultimate and time-sensitive goal in combating the COVID-19 pandemic is the development of a successful preventative vaccine. As of 25 February 2021, there are 12 SARS-CoV-2 vaccines that have been approved/authorized for full or emergency use in different areas around the world, with over 200 million doses administered worldwide [11, 184]. This experience with the development of COVID-19 vaccines has been a testimony to the outcomes that can be achieved with sufficient resources and international collaboration. Considering the trend of major coronavirus pandemics every decade so far in the twenty-first century, such international effort for an optimised and efficient emergent vaccine production plan is needed for long-term safeguarding of global health.

The current target of SARS-CoV-2 vaccines is the viral S glycoprotein. In fact, it was also the target for the development of vaccines against other coronaviruses, with attempts made in the past to develop S glycoprotein-based SARS and MERS vaccines [185, 186]. The S glycoprotein is responsible for both viral binding to the host cell receptor (ACE2 receptor), and host-viral membrane fusion for viral replication. Therefore, it is believed that S glycoprotein-based vaccines should induce the production of antibodies that block receptor binding and viral genome uncoating [187]. It has also been shown that the presence or absence of other viral glycoproteins does not affect the immunogenicity of the S glycoprotein nor its ability to bind to the ACE2 receptor, further warranting the use of this glycoprotein for vaccine development [188]. The possibility of developing a ‘pan-CoV’ vaccine is also being studied, owing to the genetic homogeneity between coronaviruses. However, it has been shown that different residues exist between SARS-CoV-1 and SARS-CoV-2, specifically in the S glycoprotein; therefore, antibodies produced against SARS-CoV-1 may not be effective against SARS-CoV-2 [189].

There are currently 6 vaccines approved for full use, and 6 others authorized for limited use against COVID-19 by various countries worldwide [190]. Additionally, there are 21 other vaccines currently in Phase III, 27 in Phase II, and 42 in Phase I [190]. Table 1 elaborates on the available details for each of the approved/authorized vaccines. They include inactivated vaccines, recombinant adenovirus (human and non-human) vaccines, and novel mRNA-based vaccines. Current studies have shown that many of these vaccines provide significant protection against severe COVID-19 (often up to 100%), and to a lesser extent, symptomatic COVID-19. Side effect profiles tend to be mild to moderate, and acute [191–202]. Both the long-term efficacy and side effects of these vaccines remain to be determined, as well as their ability to prevent transmission (sterilizing immunity).

**Table 1:** Vaccine candidates for COVID-19 as of February 25, 2021 [191–202]

Vaccine name	Organization/Institute(s)	Manufacturer	Comments
BNT162b2	–	Pfizer, BioNTech	mRNA-based vaccine 95% effective after the second dose
mRNA-1273	Kaiser Permanente Washington Health Research Institute	Moderna	mRNA-based vaccine 94.1% effective after the second dose
AZD1222 (Covishield)	University of Oxford The Jenner Institute	AstraZeneca	Chimpanzee adenovirus vaccine vector 62.1% and 90.0% effective in 2 different dosing regimens
Sputnik V	Gamalaya Research Institute of Epidemiology and Microbiology	Gamalaya Research Institute of Epidemiology and Microbiology	Recombinant adenovirus vaccine 91.6% effective after the first dose
CoronaVac	Sinovac Research and Development Co. Ltd	Sinovac	Formalin-inactivated and alum-adjuvanted vaccine
Ad5-nCoV	Tongji Hospital in Wuhan, China	CanSino Biologics	Recombinant vaccine incorporating adenovirus type 5 vector
JNJ-78436735	Janssen Vaccines and Prevention B.V	Janssen Vaccines and Prevention B.V	Non-replicating viral vector vaccine
BBIBP-CoRV	Henan Provincial Center for Disease Control and Prevention	Beijing Institute of Biological Products, and Sinopharm	Inactivated vaccine
EpiVacCorona	Federal Budgetary Research Institution State Research Center of Virology and Biotechnology	–	Peptide vaccine
Covaxin	–	Bharat Biotech, National Institute of Virology	Inactivated vaccine
CoviVac	–	Chumakov Federal Scientific Center for Research and Development of Immune and Biological Products	Inactivated vaccine
–	Wuhan Institute of Biological Products	Sinopharm	Inactivated vaccine

## Novel potential therapies

As discussed, current management of COVID-19 is supportive, with respiratory failure from ARDS being the leading cause of mortality [203]. Although the clinical safety of older medications has been established, including safety profile, side effects, physiology, and drug interactions, some medications may cause serious adverse reactions, both known and unclear, in patients with COVID-19 [203].

During the viral infection process—including intracellular transport, proliferation, and assembling of virions in the infected cell—structural and functional proteins, as well as some proteases, play a key part in the virus’s pathogenesis, suggesting that targeted-therapies against SARS-CoV-2 infection could be a promising strategy. Some drugs have displayed potent inhibitory effects on the virus in vitro and in vivo; however, not all mechanisms are clear [203]. Considering the seriousness and suddenness of the pandemic, over 200 clinical trials on COVID-19 had commenced in China alone a couple months into the reporting of the outbreak, and have successfully reported that certain targets and their agents have displayed strong antiviral potential, of which some have been permitted to be used in clinical trials [204].

Scientists have suggested multiple existing compounds to undergo clinical trials to determine their efficacy against COVID-19. The international “SOLIDARITY” trial, developed by the WHO, had set to test the efficacy of five different treatment modalities: (1) standard of care, (2) standard of care plus Remdesivir, (3) standard of care plus Lopinavir and Ritonavir, (4) standard of care plus Lopinavir, Ritonavir and Interferon beta, and (5) standard of care plus Hydroxychloroquine [205]. On the other hand, the UK’s national Randomized Evaluation of COVID-19 Therapy (RECOVERY) Trial encompassed several primary and branching treatment arms in addition to standard of care; these include the use of Lopinavir and Ritonavir, Azithromycin, low-dose corticosteroids, convalescent plasma, and Tocilizumab [206]. Furthermore, the US National Institute of Health (NIH) launched the Accelerating COVID-19 Therapeutic Interventions and Vaccines (ACTIV) trial, which is set to test in outpatient and inpatients settings, various immune modulators, monoclonal antibodies, antithrombotics, anti-retrovirals, and convalescent plasma [207]. Data has also emerged from independent studies and trials worldwide, which have contributed to the developing interim clinical consensus.

### Dexamethasone

Low-dose Dexamethasone, a potent steroid, is currently the most promising potential therapeutic for severe COVID-19. In the RECOVERY trial, 2104 patients were randomized to receive dexamethasone for ten days and compared with 4321 patients randomised to usual care alone. Dexamethasone was found to reduce deaths by one-third in ventilated patients and by one fifth in patients receiving oxygen only. However, there were no statistically significant benefits among patients who did not require respiratory support [208]—A point that needs to be stressed in order to avert cases of self-treatment, over-treatment, and drug-shortage. These findings have encouraged the WHO, NIH, and CDC to recommend the use of dexamethasone or alternative glucocorticoids/corticosteroids such as hydrocortisone where appropriate [209, 210].

Furthermore, a meta-analysis was conducted on 7 randomized clinical trials in 12 different countries, evaluating the efficacy of corticosteroids in 1703 critically ill patients with COVID-19. The meta-analysis showed that dexamethasone reduced 28-day mortality compared to standard of care or placebo by 36%. On the other hand, hydrocortisone and methylprednisolone did not significantly reduce mortality [211]. Similarly, a large systematic review and network meta-analysis on 85 trials enrolling 41,669 COVID-19 patients found that corticosteroids were the only therapeutic to reduce mortality and morbidity (mechanical ventilation) to a moderate extent compared to standard of care—A finding that did not similarly transfer to remdesivir, azithromycin, hydroxychloroquine, lopinavir/ritonavir, interferon-beta, or tocilizumab [212].

The WHO’s living guidance on COVID-19 therapeutics, developed in partnership with Magic Evidence Ecosystem Foundation (MAGIC), is based on a current systematic review and network analysis of all relevant trials. The results report lower mortality rates in critical or severe COVID-19 patients who are on corticosteroids (specifically, dexamethasone), as well as increased hyperglycaemia. The analysis includes tens of trials with an evidence quality of “low” to “moderate”. Thus, the use of dexamethasone in severe/critical COVID-19 patients is “strongly” recommended. On the other hand, due to “low quality” data showing increased mortality in non-severe cases of COVID-19 taking corticosteroids, it is “weakly” recommended against [213].

As such, dexamethasone seems to be a reasonable therapeutic for severe and critical COVID-19 patients who require supplemental oxygenation, both invasive and non-invasive [213, 214].

### Remdesivir

Remdesivir (GS-5734), an experimental intravenous drug originally developed for the treatment of Ebola virus, inhibits viral replication by inhibiting RNA-dependent RNA polymerase [215]. Notably, Remdesivir has demonstrated antiviral activity in treating MERS and SARS [216]. The first COVID-19 patient diagnosed in the United States—A young man in Washington—was given Remdesivir when his condition worsened; he improved the next day, according to a case report in the *New England Journal of Medicine* [217]. The drug has since then been tested in a number of RCTs globally. Remdesivir is currently the only antiviral drug that the CDC does not recommend against using [218]. It is recommended by the NIH either as monotherapy or with dexamethasone, in cases of hospitalized patients who may or may not require supplementary oxygenation [214]. WHO guidelines however do not find there to be enough evidence as of now to recommend its use [213].

The first double-blind randomized trial conducted with Remdesivir (n = 158) versus placebo (n = 79) in severe COVID-19 patients found no significant difference in primary outcome of time to clinical improvement within 28-days either in the intention-to-treat analysis or the per-protocol analysis. Clinically speaking however, the results slightly favoured Remdesivir over placebo in both analyses [219].

In another RCT, the National Institute of Allergy and Infectious Diseases announced the interim results of their Adaptive COVID-19 Treatment Trial (ACTT; NCT04280705)—A phase 3, randomized, double-blind, placebo-controlled trial. The trial involved 1062 patients, and was conducted at 68 sites in the USA, Europe, and Asia. Preliminary results suggested that patients treated with Remdesivir had a 31% faster time to recovery (11 days vs 15 days) than those who received placebo (p < 0.001). However, the survival benefit of Remdesivir (8.0% mortality rate) was not statistically significant compared to the placebo group (11.6%; p = 0.059) [220]. Recent update from the first stage ACTT-1 further suggests benefits for the use of remdesivir in the setting of COVID-19. The trial, which assigned 541 patients to treatment and 521 to placebo, reported a shorter median recovery time (10 vs 15 days) in patients who received remdesivir (rate ratio for recovery, 1.29; 95% CI 1.12 to 1.49; P < 0.001). The Kaplan–Meier estimates of mortality were also lower for the treatment group, with a hazard ratio of 0.73 (95% CI 0.52 to 1.03) [221].

Furthermore, the SIMPLE trial; an open-label, randomized, phase III trial in 15 countries primarily compared clinical improvement of 5-day versus 10-day treatment duration of Remdesivir in addition to standard of care, in hospitalised patients with severe COVID-19 (n = 397). The study reported similar outcomes between the 5-day and the 10-day treatment course, which, interestingly, was slightly in favor of the 5-day course. An exploratory analysis of the data, using pooled data from both arms, found that more patients were discharged earlier when Remdesivir was started early within 10 days of symptoms onset [222].

In contrast to the mentioned evidence, recent reports from the WHO SOLIDARITY Trial suggests a lack of benefit for Remdesivir. A total of 405 hospitals in 30 countries participated, with a total of 11,266 randomized adults, 2750 of which were allocated to Remdesivir. A total of 301/2743 (10.97%) patients expired on Remdesivir, compared to 303/2708 (11.1%) from the control. The death rate ratio or relative risk for Remdesivir was therefore 0.95 (0.81–1.11, p = 0.50), suggesting a lack of benefit or hazard. The preprint also reports a meta-analysis that combines data from 4 trials: SOLIDARITY, ACTT-1, and two smaller trials; the Remdesivir versus control death rate ratio or relative risk was insignificant, at 0.91 (95% CI 0.79–1.05) [223].

As for the WHO’s living guidelines on COVID-19 therapeutics, based on MAGIC’s meta-analysis, the results reported no “important difference” in any clinical outcome, including mortality, requirement and duration of mechanical ventilation, and serious adverse events. All evidence quality was classified as “low” or “very low”, concluding with a “weak” recommendation against use of Remdesivir at any COVID-19 severity.

In the absence of further evidence, Remdesivir remains a promising experimental drug in comparison to other investigated therapeutics, with at most a moderate clinical benefit. However, considering concerns of limited availability, it has been recommended in light of the recent evidence that treatment should be prioritized for hospitalized patients requiring low-flow supplemental oxygen, as they seem to derive the most benefit [224].

### Tocilizumab (IL-6 antagonists)

Tocilizumab is a recombinant humanized monoclonal antibody that targets interleukin 6 (IL-6); a pro-inflammatory cytokine that induces acute phase reactants (e.g. CRP) [225], and is highly implicated in the resultant cytokine storm. Since cytokine storms have been established as an important pathogenic mechanism of mortality in severe COVID-19 [226], the blocking of IL-6 activity may offer a promising therapeutic target in severe COVID-19.

One retrospective observational cohort study on 544 adults with severe COVID-19 pneumonia compared a non-randomly selected subset of patients who received tocilizumab in addition to standard of care (n = 179), with the rest of the controls (n = 365). The study found that after adjustment for potential confounding factors, tocilizumab treatment was associated with reduced risk of invasive mechanical ventilation or death (adjusted hazard ratio: 0.61, 95% CI 0.40–0.92; p = 0.02) [227].

The UK RECOVERY trial tested Tocilizumab in admitted patients with COVID-19, adopting a randomized, controlled, open-label, platform design. The study found that patients allocated to tocilizumab were more likely to be discharged alive within 28 days compared to standard of care (54% vs. 47%; rate ratio 1·22; 95% CI 1·12–1·34; p < 0·0001). Additionally, among patients not on invasive mechanical ventilation at baseline, those allocated to tocilizumab were less likely to reach composite endpoints of invasive mechanical ventilation or death (33% vs 38%, risk ratio: 0.85; 95% CI 0.78–0.93) [228].

Finally, In the international, multifactorial, adaptive platform trial REMAP-CAP (NCT02735707), both tocilizumab and sarilumab (another IL-6 inhibitor), met predefined criteria for efficacy against COVID-19 in critically ill patients receiving organ support in ICU. An analysis of 90-day survival showed improved survival in the pooled IL-6 receptor antagonist groups (n = 414). When compared to control group (412), patients receiving Il-6 antagonists had lower median organ support-free days. The in-hospital mortality in the pooled Il-6 antagonist groups was lower than the control group (27% vs 36%)—Median adjusted odds ratio for in-hospital survival in the tocilizumab group was 1.64 (95% CI 1.14 to 2.35), and 2.01 (95% CI 1.18 to 4.71) for sarilumab as compared to control [229].

In earlier reported trials, a clear benefit was not similarly observed in the primary outcome [230–232]. In fact, in one open-label RCT, it was suggested that tocilizumab might even increase mortality and the study was stopped early based on the interim analysis [233].

As such, the US CDC’s treatment guidelines now recommend the use of tocilizumab in combination with dexamethasone in certain hospitalized patients who exhibit rapid respiratory decompensation due to COVID-19. Based on the results of the RECOVERY and REMAP-CAP trials, these patients should be either (1) recently hospitalized patients who were admitted to the ICU within the prior 24 h, requiring invasive or non-invasive ventilation, or HFNC, or (2) recently hospitalized patients not in the ICU with rapidly increasing oxygen demands (requiring HFNC or non-invasive ventilation) and have significantly increased inflammatory markers [234].

### Anti-SARS-CoV-2 monoclonal antibodies

Monoclonal antibodies, currently undergoing initial stages of testing, have been developed against SARS-CoV-2’s virulence factors. The most prominent of these tests is the Blocking Viral Attachment and Cell Entry with SARS-CoV-2 Neutralizing Antibodies (BLAZE-1) trial, targeting various components of the virus’s spike glycoprotein and cell entry mechanisms. 533 patients were included in the final analysis of Phase II of the study, randomized to three main groups: Bamlanivimab monotherapy (700, 2800, and 7000 mg), combination treatment group (bamlanivimab and etesevimab), or placebo. Compared to placebo, the difference in the change in log viral load at day 11 from baseline was only significant for the combination group. As for secondary outcome measures (symptom relief and clinical progression), each treatment group had statistically significant differences in outcome for 10 out of 82 of these endpoints. These findings however were restricted to non-hospitalised patients with mild to moderate COVID-19 illness [154].

Yet-to-be-published results from Phase III of the BLAZE-1 trial randomized 1,035 participants with mild to moderate COVID-19 (but a high risk for disease progression) to either the bamlanivimab plus etesevimab arm (n = 518) or to the placebo arm (n = 517). The study found that participants who received bamlanivimab plus etesevimab as opposed to placebo had a 5% absolute reduction and 70% relative reduction in risk for COVID-19 related hospitalisation or death from any cause (p < 0.001). Additionally, there were no deaths in the intervention arm, compared to 10 deaths in the placebo arm (p < 0.001). Virus level decline was also greater and more rapid in the group that received the combination antibody therapy as opposed to placebo [234].

A Phase I/II randomized trial comparing a combination of casirivimab plus imdevimab to placebo has also been conducted. Interim analysis suggests potential clinical benefit from the combination therapy for outpatients with mild to moderate COVID-19, who receive the drug infusion a median of 3 days after symptom onset. In terms of outcomes, 2% (8/434) of participants in the pooled casirivimab plus imdevimab arm, as opposed to 4% (10/231) in the placebo arm, were hospitalised or required emergency department visits within 28 days of treatment. In those specifically at higher risk for hospitalisation, 3% (4/151) in the combination therapy arm as opposed to 9% (7/78) in the placebo arm were hospitalised or required emergency department visits [235].

As of recent, the US FDA issued emergency use authorization for the use of investigational monoclonal antibody therapy bamlanivimab for the treatment of mild-to-moderate COVID-19 in adult and pediatric out-patients [236]. Likewise, the US NIH echoed these recommendations, stressing on its use for those at increased risk for disease progression [234].

### Hydroxychloroquine

Hydroxychloroquine for a while had been the drug of choice for large-scale use before the emergence of controversial findings, due to its availability, safety record in Malaria patients, and relatively low cost [237]. Chloroquine and its derivatives, including hydroxychloroquine and chloroquine phosphate, have elicited antiviral effects on several viruses such as SARS-CoV and Human Coronavirus 229E by interfering with endosomal acidification [238]. Based on the advantage of known broad-spectrum activity and supposed safe adverse effects profile, a series of RCTs on chloroquine and its derivatives for COVID-19 treatment advanced rapidly. Therapeutic effects were observed in aspects of fever reduction, improvements on CT imaging, and disease progression [238]. In light of the preliminary clinical data, chloroquine had been added to the list of trial drugs in the Guidelines for the Diagnosis and Treatment of COVID-19 published by National Health Commission of the People’s Republic of China [237].

Initially, Hydroxychloroquine seemed to be a promising drug in early small trials. An open label non-randomized clinical trial conducted in France set out to test the effects of Hydroxychloroquine and azithromycin. In the study, a total of twenty COVID-19 positive patients received 600 mg of hydroxychloroquine daily, and their viral load measured on a daily basis in a hospital setting. Depending on the patients’ clinical presentation, azithromycin was added to their treatment; a significant reduction in the viral load at day 6 post Azihromycin inclusion compared to control group was observed. Additionally, in patients who had Azithromycin added to Hydroxychloroquine, a synergistic effect was reported [239]. As a single arm study nonetheless, this may have been the normal course of the disease in this small sample size, allowing much room for bias. Another study, a randomized parallel-group trial (n = 62), suggested the use of hydroxychloroquine could shorten time to clinical recovery (body temperature and cough), and improve pneumonia (ChiCTR2000029559) [240]. On the other hand, a multicenter, open-label, randomized controlled trial (n = 150) found that the administration of hydroxychloroquine with standard of care did not result in a significantly higher probability of negative conversion by day 28 (two negative PCR tests 24 h apart) than standard of care alone in patients hospitalized with persistent mild to moderate COVID-19. Additionally, adverse events were higher in hydroxychloroquine recipients (ChiCTR2000029868) [241].

Overall, there has been much controversy with regards to the use of Hydroxychloroquine in both scientific and public discourse. The WHO halted the SOLIDARITY trial’s Hydroxychloroquine arm following a retrospective observational analysis published in *The Lancet* that suggested an association with increased mortality [242, 243]. The paper was later retracted due to data integrity issues, following announcement of resumption of WHO’s hydroxychloroquine arm of the SOLIDARITY trial on the basis of the available interim mortality data [244].

Hydroxychloroquine’s adverse event profile in healthy has also been looked at in the HyPE study. In a retrospective, cross-sectional, web-based survey, data was collected on COVID-19 negative and asymptomatic healthcare workers (n = 166) who were taking hydroxychloroquine prophylactically. Overall, a higher incidence of adverse events was reported (37.9%) compared to data from studies of patients on long-term hydroxychloroquine therapy, with gastrointestinal bleeding being the most common. This finding was more prominent in those under 40 years of age. The self-reported nature of this study remains a limitation [245].

In line with the growing negative attitudes towards Hydroxychloroquine, the RECOVERY (NCT04381936) trial found no significant difference in the primary endpoint of 28-day mortality, or any evidence of beneficial effects on hospital stay duration or other outcomes, in patients randomised to Hydroxychloroquine (n = 1542) vs usual care alone (n = 3132) [246]. In another randomized, double-blind, placebo-controlled trial (n = 423), the use of Hydroxychloroquine in non-hospitalised adults 4-days within symptom onset, did not substantially reduce symptom severity, but did significantly increase prevalence of adverse events [247].

Finally, the WHO SOLIDARITY trial reported that 104 (10.98%) of 947 patients on hydroxychloroquine had expired, compared to 84 (9.27%) of 906 controlled. The relative risk or death rate ratio was therefore 1.19 (0.89–1.59, p = 0.23)—The highest out of all the other investigated drugs in the trial [223]. As for the WHO’s living guidelines on COVID-19 therapeutics, based on MAGIC’s meta-analysis, the results reported no “important difference” in any clinical outcome, including mortality, requirement for mechanical ventilation, admission to hospital, and viral clearance at seven days. However, there were fewer cases of diarrhoea and nausea/vomiting reported in supportive care as opposed to hydroxychrloquine arm. All evidence quality was classified ranged from “very low” to “moderate”, concluding with a “strong” recommendation against use of Hydroxychloroquine for COVID-19 patients at any severity [213].

In view of the emerging evidence, the FDA revoked Hydroxychloroquine and Chloroquine’s emergency use authorization to treat COVID-19 in certain hospitalized patients, unless a justifiable clinical trial is available and participation is feasible [248]. Several large trials have also been halted globally, including that of the US National Institute of Health [249].

### Lopinavir and ritonavir

This drug combination, sold under the brand name Kaletra, was approved in the US in 2000 to treat HIV infections. Lopinavir specifically inhibits HIV protease, an important enzyme that cleaves a long protein chain into peptides during the assembly of new viruses. Since Lopinavir is readily broken down in the human body by our own proteases, it is given with low levels of Ritonavir, another protease inhibitor, that prolongs the effects caused by the action of Lopinavir. This combination has been shown to inhibit the protease of other viruses as well in-vitro, specifically coronaviruses [250].

Lopinavir and Ritonavir were investigated for their potential to treat patients with SARS in China in 2003. Furthermore, shortly after the emergence of MERS, researchers also identified Lopinavir and Ritonavir as inhibitors of MERS-CoV [237]. However, the first trial of Lopinavir and Ritonavir to treat COVD-19 was not encouraging [251]. This trial was an open-label, individually randomized, controlled trial, conducted in early 2020 in Wuhan, China. Of the 199 patients who underwent randomization, 99 patients were assigned to the treatment group with Lopinavir and Ritonavir twice a day for 14 days, in addition to standard care, and 100 patients to the control group with standard care alone. Patients assigned to the Lopinavir and Ritonavir treatment group did not have a time to clinical improvement that differed from that of the patients assigned to standard care alone in the intention-to-treat population [251]. Additionally, it was determined that the viral RNA loads over time did not differ between the Lopinavir and Ritonavir recipients and those receiving standard care. Although treatment with Lopinavir and Ritonavir did not significantly accelerate clinical improvement, reduce mortality, or diminish throat viral RNA detectability in patients with serious COVID-19 in this study, it is important to note that both groups were heterogeneous and received various additional treatments, including other pharmacologic interventions such as interferon (11%) and glucocorticoids (34%) [252]. However, the median time from symptom initiation was 13 days, which may not be ideal to identify a difference between groups, specifically that the study was underpowered (recruitment was suspended early due to Remdesivir being available for clinical trials). Additionally, the recruited patients had more severe illness [251]—It is known to be questionable whether antivirals would have a significant role in later disease stages.

On the other hand, in a systematic review and meta-analysis of the efficacy and safety of antiviral treatments for COVID-19, Lopinavir-Ritonavir combination was the only positive outcome with “low-quality evidence” suggesting a small decrease in mortality and reduction in length of hospital and ICU stay for severe COVID-19, in addition to “moderate-quality evidence” suggesting likely increases in diarrhea, nausea and vomiting. The other drugs, including Hydroxychloroquine, Ribavarin, Interferon, Umifenovir, and Favipiravir, were only met with “very low-quality evidence” with little or no suggestion of benefit for most treatments and outcomes in both non-severe and severe COVID-19 [253].

The WHO SOLIDARITY trial recently reported that the relative risk of Lopinavir (co-administered with Ritonavir) was 1.00 (0.79–1.25, p = 0.97) with a mortality of 148/1399 (10.58%), compared to 146/1372 (10.64%) in the control group. Furthermore, the joint mortality combining SOLIDARITY, RECOVERY, and other smaller trials was 1.02 (95% CI 0.91–1.14) [223]. As such, the use of these agents in COVID-19 patients is not supported by the current evidence.

As for the WHO’s living guidelines on COVID-19 therapeutics, based on MAGIC’s meta-analysis, the results reported no “important difference” in any clinical outcome, including mortality, requirement for mechanical ventilation, admission to hospital, and viral clearance at seven days. However, similarly to hydroxychloroquine, there were fewer cases of diarrhoea and nausea/vomiting reported in supportive care as opposed to the lopinavir-ritonavir arm. All evidence quality was classified ranged from “very low” to “moderate”, concluding with a “strong” recommendation against use of lopinavir-ritonavir for COVID-19 patients at any severity [213].

### Interferons

Interferons (IFNs) are cytokine proteins that bind to cell surface receptors and initiate signalling cascades, which have shown to be effective against many viruses like Hepatitis B and Hepatitis C [254]. Studies evaluating the antiviral activity of types I and II interferons have reported that interferon beta is the most potent interferon in reducing in vitro MERS-CoV replication [254]. A combination of these drugs are now being tested on MERS patients in Riyadh, Saudi Arabia, in the placebo-controlled MIRACLE trial [255]. The study assesses the feasibility, efficacy and safety of a combination of Lopinavir/Ritonavir and Interferon Beta-1b in hospitalized patients with MERS [256].

The efficacy of this combination with interferon alpha was analysed in a retrospective cross-sectional study from two hospitals in Anhui, China, on 181 patients with confirmed COVID-19. The analyses suggested that early initiation of lopinavir/ritonavir plus IFN‐α combination therapy was associated with a shortened duration of SARS‐CoV‐2 RNA shedding (HR 1.649 [95% CI 1.162–2.339] [257].

In another RCT (IRCT20100228003449N28) on the efficacy and safety of Interferon Beta-1a in treating severe COVID-19, a total of 42 patients were randomized into the IFN group and 39 patients into the control group. Time to clinical response was not significantly difference between both groups. However, more patients in the IFN group were discharged on day 14 compared to the control group (odds ratio = 2.5; 95% CI 1.05 to 6.37). Additionally, the 28-day overall mortality was significantly lower in the IFN group (19%) vs control (43.6%, p = 0.015). Early administration was also found to significantly reduce mortality [258].

Treatment with nebulized IFN-α2b has been shown to be promising in a retrospective study of 77 hospitalised patients with COVID-19. The study showcased a significantly reduced duration of detectable virus in the upper respiratory tract and a parallel reduction in duration of elevated blood levels for inflammatory markers IL-6 and CRP. This remained true when IFN-α2b was administered with or without arbidol [259].

However, several of the studies mentioned suffer from methodological limitations and relatively small sample sizes. On the other hand, the WHO’s SOLIDARITY trial reported based on about 4000 patients, that the mortality relative risk for IFN Beta-1a with Lopinavir co-administration was 1.16 (95% CI 0.96–1.39, p = 0.11), and 1.12 (95% CI 0.83–1.51) without Lopinavir co-administration; all of which point to lack of significant benefit. As such, the current evidence for the use of these Interferons in the treatment of COVID-19 is not sufficient and is recommended against by the US CDC unless in the context of a clinical trial [260].

### Convalescent plasma

Treatment via convalescent plasma has also attracted some attention, with several clinical trials currently recruiting [261]. A retrospective, propensity score-matched, case–control study that assessed convalescent plasma therapy in 39 patients with severe or life-threatening COVID-19 reported improved oxygen requirements, and survival [262]. One meta-analysis of three clinical studies for COVID‐19 in China, showed a statistically significant improvement in clinical outcomes of patients treated with convalescent plasma (n = 19) compared with historical controls (n = 10; P < 0.001) [263].

An RCT of COVID-19 severe pneumonia assigned 228 patients to receive convalescent plasma and 105 to receive placebo. Overall mortality was 10.96% in the intervention arm and 11.43% in the control group, which was not statistically significant. No difference was noted in the distribution of clinical outcomes according to a 6-point ordinal scale on day 30 either. SARS-CoV-2 antibodies titres however tended to be higher in the convalescent plasma group at day 2 after intervention, with similar adverse events in both groups [264].

A living Cochrane systematic review (n = 38,160 participants of whom 36,081 received plasma) reports uncertainty regarding convalescent plasma’s ability to decrease all-cause mortality, and little to no difference in improvement of clinical symptoms [265]. Potentially associated unwanted effects however, also with low evidence, include death, allergic reactions, thrombotic or cardiac events, and respiratory complications [265]. Blood clotting (due to residually active pro-coagulant factors in transfused convalescent plasma) has especially been brought up as a concern, since COVID-19 patients are considered at increased risk [266]. The evidence supporting this however remains low, as iterated by the Cochrane review.

Note that another meta-analysis and systematic review (n = 35,055) reported that aggregation of mortality data from all controlled studies, including RCTs and matched-controls, indicated that patients transfused with convalescent plasma exhibited 42% reduction in mortality rate compared to patients receiving standard treatment (20% vs 28%; OR: 0.58, 95% CI 0.47–0.71, P < 0.001). Furthermore, an additional dose–response analysis found that the aggregate mortality rate of COVID-19 patients transfused early-on with higher-titre convalescent plasma was lesser than that of patients transfused with lower titre.

Overall, more information will be needed from clinical trials before recommending this approach, thus remaining as a last resort in compassionate use.

## Global health response

**Fig. 4:**
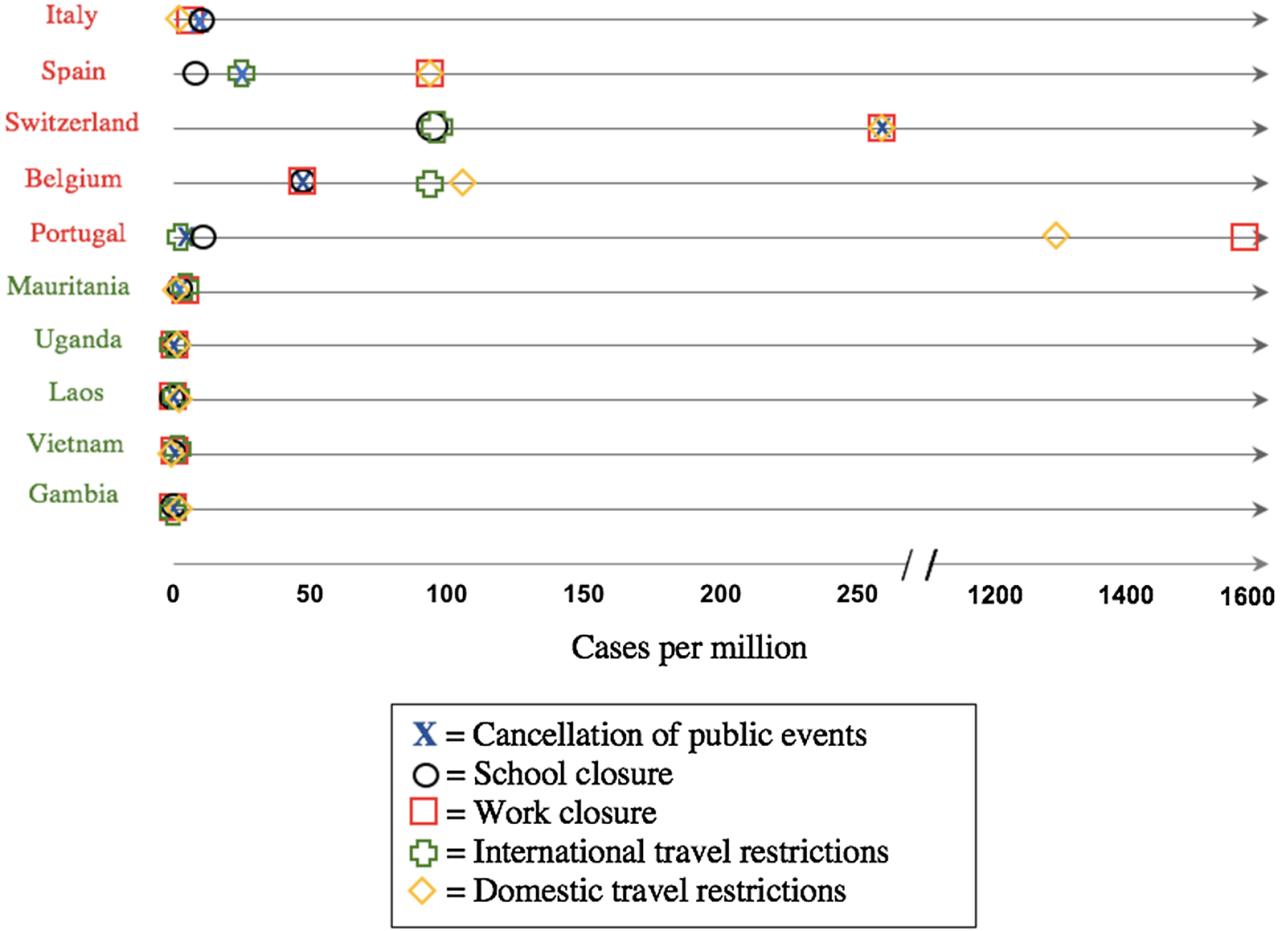
Comparison between the number of COVID-19 cases per million when public health containment initiatives were taken by the five countries (Mauritania, Uganda, Laos, Vietnam, and Gambia) with the lowest number of cases per million and the five countries (Italy, Spain, Switzerland, Belgium, Portugal) with the largest number of cases per million, in the first 30 days since their first confirmed case [11, 272]. Countries with a population of less than one million or with exceptional circumstances (civil war) were excluded [11, 272]

The global response to the COVID-19 pandemic has widely varied, including complete lockdowns, social distancing measures, and population screening policies—or none of the above (Fig. 4). The outbreak continues to exert pressure beyond capacity on countries globally, revealing in some instances a lack of preparation and infrastructure to protect the public and healthcare practitioners, as was seen by the shortage in emergency medical supplies [267]. COVID-19 has proven to be difficult to control as compared to previous outbreaks due to a large number of cluster transmissions or superspreader events, relatively limited health resources, and the unavailability of rapid testing kits [268, 269]. As seen in Fig. 4, countries that enforced public health measures early on during the progression of their national outbreak, were better able to control the spread of the virus compared to other countries who had not done so. Additionally, vaccine roll-out responses have been widely variable. Several countries, such as China, Russia, India, the US, and the UK, have been directly involved with the production of vaccines [190]. Multiple other countries have instead led randomized controlled trials testing their safety and efficacy. In terms of vaccination rates, as of February 25, 2021, Israel, the United Arab Emirates, US, UK, and Chile have had the highest total number of vaccination doses per 100 people [270]. However, this list continues to vary throughout the pandemic. Global equitable access to vaccines has also been a major concern, which propelled the WHO’s COVAX initiative for accelerated equitable access to vaccines worldwide [271].

### China and the border Asian region

On December 1, 2019, the first symptomatic patient was identified with SARS-CoV-2 in the Huanan Seafood Market in Wuhan of the Hubei province in China; the epicentre of the pandemic [6]. On January 23, 2020, weeks after SARS-CoV-2 was identified, the Hubei province underwent a lockdown. Other provinces followed suit on February 11, 2020 due to an increase in the number of cases nationally [273, 274], which began to decline on March 15, 2020 [275]. The lockdown on Wuhan is theorised to have delayed the spread to other areas in China by 2.91 days and decreased the number of cases by 33.3%. Additionally, it is thought to have reduced worldwide spread by 77% with a two to three weeks delay in the spread [273, 276].

Other areas in the Asian region responded quickly, using strategies that were refined after the 2003 SARS and 2009 H1N1 Influenza outbreaks. South Korea responded by distribution of test kits early on, by February 7, and implementing restrictive measures by February 23, a month after their first case. Additionally, they established 600 screening sites nationally [11, 277]. South Korea’s CFR as of February 25, 2020 is 1.8% [11]. Taiwan also increased its laboratory capacity by building a national program to include 27 laboratories in the country, and currently boasts a CFR of 1.04% [11, 278]. Meanwhile, Singapore announced an orange alert 15 days after their first case. In January 2021, they announced the use of the Moderna vaccines, in addition to implementing tighter restrictions on travels from South Africa after reports of a new variant [279]. As of February 25, 2021, their CFR is less than 0.01% [11]. Hong Kong, on the other hand, responded before the appearance of their first case [280, 281]. It is noteworthy that these countries have an elderly population that forms only 10 to 14% of the country, which may have contributed to their greater success in containing COVID-19 when compared to other countries [269].

Nonetheless, Japan, which has the largest elderly population (26%), boasted a relatively low number of cases in comparison to Italy, which has the second largest elderly population (23%). The reason behind the difference between Japan and Italy’s total number of cases is yet to be determined but has been theorised to be due to a lower frequency of testing [282]. Due to Japan’s initially limited testing capacities, the authorities had opted to depend on mitigation measures [283, 284]. However, on January 19, 2021, Japan launched a COVID-19 Robot testing system, and began mass random PCR testing in cities [285]. As such, on February 25, 2021, Japan had done 60.31 tests per 1000 people [11]. Japan has currently implemented a state of emergency starting from February 2, 2021 up until March 7, 2021 in order to mitigate the “3rd wave” of COVID-19 that began in November 2020 [285, 286].

As of February 2021, the countries with the highest cases in the region are India, Indonesia and Sri Lanka [285, 287]. The region as a whole, excluding China, houses 16.99% of global COVID-19 cases, and 18.06% of the global deaths [11, 287, 288].

### Middle East and North Africa region

The majority of the region implemented mitigation strategies, as described below [289]. Iran, the epicentre of the region, began its efforts against COVID-19 on February 19, 2020 with the formation of the COVID-19 National Committee. Partial restrictions were enforced, such as cancellation of congregational prayers. Neighbouring countries also suspended flights to and from Iran on February 25, 2020 [31, 290]. As of February 2021, Iran has closed all schools, placed a stricter travel ban and a night traffic ban. They have also introduced a national vaccine campaign [291]. Additionally, Saudi Arabia began taking actions before their first case with the suspension of pilgrimage visits [11, 292]. Following a short-lived return to normal by July of 2020, a lockdown was brought back in February 2021 to suppress a rise in cases [293]. Similarly, Jordan enforced one of the strictest complete lockdowns globally [294]. On October 1, 2020, schools and universities were shut down due to a cluster of cases linked to the student population. By February 7, 2021, schools had gradually begun re-opening. Additionally, Jordan was the first country in the world to begin vaccinating refugees and asylum seekers in its territories [295].

The Eastern Mediterranean Region makes up 6% of cumulative cases worldwide, and 6% of the deaths overall [287]. Under-testing and lack of funding has been one of the major points of struggle. Many countries, such as Iraq, attempted to increase their testing by opening new laboratories [296]. The WHO’s regional office has also received support through an increase in PPE and laboratory supplies in Dubai and other countries. Financially, $71 million in funds has been secured (Kuwait—$41 M; Saudi Arabia—$10 M) [289, 292, 297]. The WHO has also donated over 55 tons of health supplies to Syria [298]. Another issue that has surfaced in the region is the spread of COVID-19 among migrant workers’ camps, as seen in Bahrain, and the wider GCC. The public health policies have been widely dynamic, changing throughout the pandemic as new evidence appeared. Countries like Bahrain for instance cancelled their mandatory 10-day quarantine and tracing bracelets for all travellers, as only 0.2% of arrivals were positive after the 10 days, which was considered not significant enough to continue the measure [299]. Bahrain then began a nationwide vaccination program, which placed it as one of the top three internationally throughout the period of December 2020 and January 2021in terms of population vaccination rates [300].

### Europe and the UK

During March of 2020, Europe became the global epicentre of COVID-19 cases, and only began to see a reduction in its cases around June 2020. As of September 22, 2020, Europe made up 14.38% of cases worldwide, ranking fourth out of the continents [11, 298]. The situation began in Italy, which rapidly deteriorated starting from January 23, 2020, leading to a relatively high CFR. On January 30, all travels to China were banned, followed by severe mitigation policies (National Red Zone) which were put in place from March to May 2020. By February 21, 2020, Travellers departing from Italy had spread COVID-19 to 21 other countries [301]. Italy began its reopening phase around May, 2020, easing out the several month-long restrictions. [302] Different areas in Italy seemed to report varying CFRs; studying the different containment strategies in each area and their correlation, if any, with the reported CFR, would be worthwhile [301]. As of February 25, 2021, Italy had a cumulative testing rate of 648.8 tests per thousand people, bringing down its CFR from 14.4% during September of 2020, to 3.4% as of February, 2021 [11, 303].

As of February 21, 2021, Europe ranks second (34%) after North America (45%) in percentage of cumulative cases [287]. The CFR of different countries in this region greatly vary, with Iceland having the lowest CFR (0.5%) and Bulgaria the highest (4.1%). Other countries fall in between this range, such as Germany with 2.9%, and Italy with 3.4% [11].The difference between the CFRs can be attributed to many factors. In terms of age, an established co-morbidity, a comparison between the average age of the populations in Germany (46 years) and Italy (63 years) may point towards a correlation. Another potential factor is the capacity of the respective healthcare systems, with Germany’s ICUs providing 29 beds per 100,000 people, compared to Italy with 12 beds per 100,000 people, and Spain with 10 beds. Additionally, the timing of the response may be a main differentiating factor, as some public health measures were enforced relatively late into the spread of COVID-19 [304]. Iceland for instance, with a relatively low CFR, had started implementing random testing (population screening) before their first confirmed case. Patients with a negative result in quarantine were re-tested, which contributed to 54% of the confirmed cases [305].

Initially, the United Kingdom (UK) opted for a herd-immunity approach; however, mitigation strategies were implemented on March 18, 2020, when the daily new COVID-19 cases reached 407 per day [11, 306, 307]. The UK, at 382.1 deaths per million cases, has exceeded Italy’s death toll at 298.1 deaths per million cases (As of February 25, 2021) [11]. However, the UK still remains lower on the scale in comparison to the USA, which is at 2,090.9 deaths per million cases. Around August of 2020, the UK began easing up lockdown measures, before resuming stricter measures on November 5, 2020, announcing an ending date of March 2021. This reactive response may have been as a result of the spike that was brought by the reopening of education institutions gradually in June. By doing so, the UK authorities risked raising the reproductive number above 1 [308]. On November 8, 2020, Scotland elevated their restriction to a level 5, meaning individuals are only allowed to go out for an emergency. Following this, a few travel restrictions were placed again, such as a 14-days quarantine for travellers coming from certain countries (e.g. Spain) starting from December 12, 2020. This continued into 2021, with travellers required to quarantine and take two NP swabs before ending the quarantine if they are arriving from COVID-19 hotspots. On January 8, 2021, the UK hits its highest number of cases per day, at 68,053, with the death toll peaking on January 20, 2021, at 1,820 death [309]. Additionally, once the UK had identified the presence of a variant of concern in the country, known as B.1.351, which originated from South Africa, the government decided that it would perform additional testing and sequencing in eight different areas in England. While this is less than one in every 10 samples from people who test positive for COVID-19, the UK is the second highest country in Europe to test and sequence the variant of COVID-19. As a result, they have carried out almost half of the COVID-19 genome sequence globally. [Wise J. Covid-19: The E484K mutation and the risks it poses.] On February 22, 2021, the government of the UK announced that they will lift all lockdown restrictions by June 21, 2021, with educational institutes reopening on March 8, 2021. In terms of testing, the UK began testing door-to-door on all households starting from February 1, 2021 [309].

### North and South America

On June 1, 2020, North America ranked first for number of COVID-19 cases and second for total death rate. The USA, encountering its first case of COVID-19 on February 26, 2020, started to reinforce testing and public health measures a month later, influenced by the severity of the predicted death count of up to 2.2 million if restrictive measures were to not be implemented. Consequently, the outbreak in late February of 2020 in Washington was not detected and mitigated in a timely manner. One of the largest set-backs that the healthcare system in the USA had faced included shortage of emergency supplies, such as masks, protective equipment and detection kits [11, 305]. However, the public health responses in the US have varied widely between different states. For instance, during the Month of March 2021, states such as California, New York, and Los Angeles, had broad public face mask requirements enforced both indoors and outdoors. States like Minnesota however enforced masks inside public buildings/businesses only. On the other hand, many other states, including Texas, Missouri, and Montana, did not have any face-mask mandates [310]. This variation extended similarly to travel restrictions and stay-at-home orders [310]. The Biden-Harris administration’s “plan to beat COVID-19” includes giving all citizens access to free testing, investing in vaccines to be distributed for free to all American citizens, and implementing a public-setting mask requirement nationwide [311]. Additionally, on January 21, 2021, the United States decided to reverse its decision to withdraw from the WHO, in order to strengthen its plan for combating COVID-19 [312]. As of February 25, 2021, the USA has a CFR of 1.8% and has performed 2.73 test per 1,000 people [11].

Canada entered a state of emergency on March 17, 2020, a week after its first case, and expanded its ICU capacities in preparation [313]. However, the authorities in Canada did not broaden the traveling restrictions accordingly nor enforced testing of all passengers on arrival; hence, out of the initial 118 cases, 30% came from Iran, 18.2% from the US and 13.1% from Europe [314]. Canada likewise faced a shortage of PPE and medical resources [315]. Canada has also remained on a strict lockdown up until June 25, 2020, before starting to ease up and reopen certain businesses. However, a few regions continued to extend their lockdowns up until October 7, 2020. On January 5, 2021, Canada enforced COVID-19 testing for all air travellers. With the easing of the restrictions, Canada’s 7-day average for new daily cases reached 9,626.86 by January 9, 2021. As a result, on January 12, 2021, Ontario imposed a stay-at-home order, permitting people to go out shopping for necessities only and for exercising. In addition, travel restrictions were placed against the US and UK. This is planned to continue until the end of March 2021 [316]. As of February 25, 2021, Canada managed to reduce the 7-day average of new daily COVID-19 cases down to an average of 2,986.43 per day, and now has a CFR of 2.5% [11].

On the other hand, South America has had varying responses throughout the continent, with Paraguay promoting strict mitigation strategies, and Chile and Colombia establishing a consistent testing policy along with other strict measures [317]. The South American region stands out due to the wide inequalities in income and the lack of equal access to healthcare services [318]. In the region, the only country that has not implemented any suppression strategies or strict policies is Brazil. This has overwhelmed their healthcare system, with a CFR that had reached 7.0% in May, dropping to 2.4% by February 25, 2021 [11]. As a continent, South America constitutes 18.4% of the cumulative confirmed deaths from COVID-19 (February 25, 2021) [11]. The biggest challenges faced in the region included limited healthcare resources, and lack of consistent compliance by the population to the public health measures in place [318]. Currently, both Brazil and Mexico place second and third within the region of America for number of cases, respectively.

### Australia and New Zealand

Australia saw its first case of COVID-19 on January 25, 2020, and implemented travel bans to China, Iran and Italy on February 1, February 29, and March 10, respectively. It is estimated that the travel ban on China reduced the potential number of cases and deaths by 87% [319]. The country has performed 54.81 tests per 1,000 people, with one confirmed case per 202.4 tests, exceeding the test ratio performed by South Korea and Iceland, both of which were considered high in their respective regions [11]. As of February 25, 2021, Australia’s CFR is at 3.1%. Since December 23, 2020, the country has been on lockdown due to the surge of cases [320].

New Zealand saw its first case on February 28, 2020 and closed its borders on March 19, 2020 with a recorded 5.81 cases per million people. The country eventually entered a strict lockdown on March 25, 2020 [321]. They have performed 191.75 tests per 1,000 people, with one confirmed case per 3,079.0 tests, placing themselves at the top for the least number of positive cases per tests by the end of September, 2020 [11]. Overall, New Zealand adopted a proactive approach and implemented strict policies early on, which may have contributed to the relatively low CFR of 1.70% [11, 321]. As of February 2021, New Zealand has managed to avoid being severally impacted by COVID-19 and has maintained an almost COVID-19-free status via adoption of an elimination strategy as opposed to mitigation and suppression [322].

### Africa

**Fig. 5:**
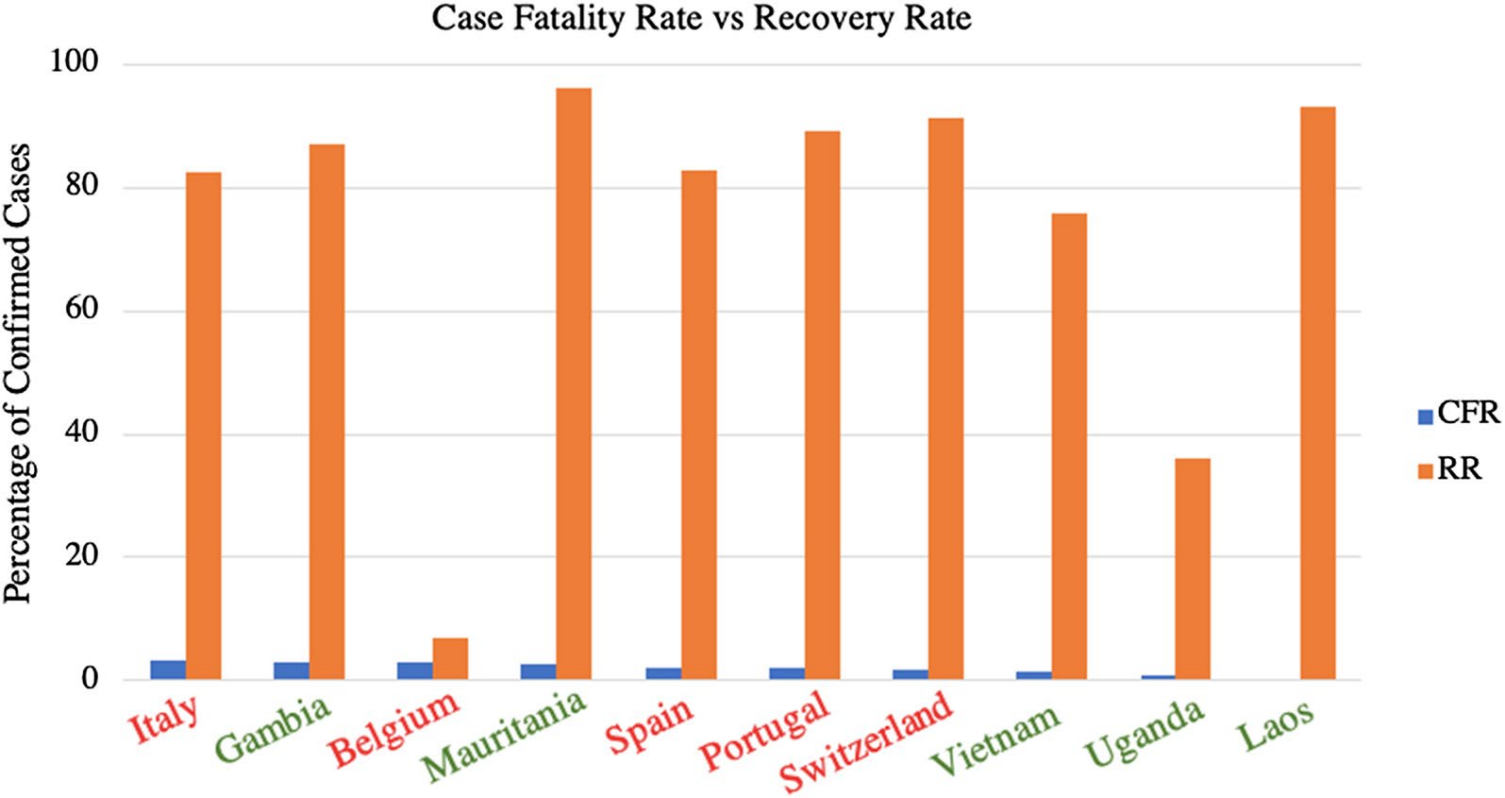
A comparison of the Case Fatality Rate (CFR) and the Recovery Rate in the five countries (Mauritania, Uganda, Laos, Vietnam, and Gambia) with the lowest number of cases per million in the first 30 days since their first confirmed case, to the five countries (Italy, Spain, Switzerland, Belgium, Portugal) with the largest number of cases per million in the first 30 days since their first confirmed case [11]

In Africa, the regional CDC began its emergency response on January 27, 2020, quickly implementing mitigation and containment measures to control any potential spread of the disease. By March 20, 2020, they were already seeing a reduction in their average daily case growth. With the extra time that the continent had, they prepared for a continent-wide response, in which they increased their labs from 2 to 43 by mid-March. Additionally, they received funding and medical supplies from various NGOs. Finally, the African Union (AU) announced that they would start a COVID-19 Respond Fund which would support Africa CDC in accelerating COVID-19 testing [318]. This has been well-reflected in the outcomes, as Africa makes up only 3% of the COVID-19 cases worldwide, and 3% of the deaths as of February 23, 2020, despite forming around 17% of the world population (Fig. 5) [11, 287].

### Innovative COVID-19 coping responses

Several forms of adaptations in various sectors have been adopted worldwide to cope with the changes brought forth by the COVID-19 pandemic. Telemedicine for instance was introduced in multiple countries to avert the risks of in-person medical visits; these include Singapore (March 8), Australia (March 11), Saudi Arabia (March 12), UK (March 17) and the USA (March 17) [323–325]. In addition, virtual education has been adopted in most countries worldwide, in order to avoid disruption of student learning after closure of campuses [326, 327]. In addition, various innovative forms of “contact-less” processes, such as payment and delivery, have been introduced by institutions to decrease risk of virus transmission [328, 329]. However, as of the end of May 2020, many countries began studying the option of re-opening and easing restrictions to varying extents, which sparked both public and scientific controversy, and continues to be a point of contention. For instance, according to Kim et al. (2020), school closures have mitigated COVID-19 spread in Korea and re-opening them could result in doubling the cases [330]. On the other hand, the efficacy of school closures as a means to reduce the number of new COVID-19 cases is considered insignificant in countries like Taiwan, due to already low transmission rate and the minimal number of new cases in the younger population [331]. Sweden has also been argued as a case against the closure of schools, considering that despite schools remaining open nationwide a low incidence of severe Covid-19 among schoolchildren was observed [332]. Nonetheless, it is important to point out that severe disease in children has regardless been reported as low in prevalence globally—As such, closure of schools is argued for in order to prevent transmission from children to the adults and elderly they are in contact with who may be more at risk for severe disease [333]. The responses to the COVID-19 pandemic have been both reactionary and proactive, with policies remaining dynamic throughout. Overall, as theoretical concepts are applied and tested in a novel setting, global health responses to COVID-19 continue to pose a challenge for all stakeholders involved, both within public and scientific circles.

## Global economic burden

The outbreak of SARS-CoV-2 has resulted in a global economic slowdown, as demonstrated by the 2020 crash of global financial markets due to the disruption of international business activities [334]. The pandemic has disrupted international trade, as the global supply chain systems used by organizations and oil-producing countries to conduct business at the global level have been terminated as a result of precautionary measures taken by states to reduce the spread of the virus. In effect, the value of international trade is deteriorating. The World Trade Organization (WTO) estimates the volume of global trade to have declined by an overall of 9.2% in 2020, due to the pandemic’s economic shock [335]. According to the UNCTAD, global trade began a strong recovery in Q4 of 2020 due to an 8% growth in goods trade [336]. The recovery of international trade is projected to stall in the first quarter of 2021 (1.5% fall in the trade of goods relative to Q4 of 2020) due to continuous disruptions in the travel sector and the trade of services caused by virus surges [336]. The UN DESA expects global trade activity to remain below pre−pandemic levels until 2022 [336]. An overall 6.9% rebound in the cross-border trade of goods and services is projected in 2021, subject to the wide rollout of vaccines, the lift of movement restrictions and uncertainties over the pandemic clearing up [337].

According to the World Bank, the global economy faced in 2020 the deepest recession since 1945, with a 4.3% contraction in global GDP and a 6.2% decline in global GDP per capita [338, 339]. The global GDP is projected to expand by 4% in 2021 and 3.8% in 2022 (global GDP remains 5.3% and 4.4% below pre-pandemic levels respectively) [338]. The cumulative estimated cost of SARS-CoV-2 on the global economic output in 2020 and 2021 is USD8.5 trillion and USD22 trillion between 2020 and 2025 [340, 341]. The economic implications of the COVID-19 outbreak and the global economic growth commence into recovery depends on the path of the virus, duration of the pandemic and the success of vaccines. In addition to agreements made by governments and pharmaceutical companies on vaccines’ distribution mechanism.283 The International Monetary Fund (IMF) anticipates that a wide rollout of effective vaccination could stimulate a 5.5% economic growth in 2021, with economic recovery rates varying across countries depending on policy response effectiveness, vaccination speed, medical interventions, monetary policy initiatives and structural economic characteristics [340]. Although, new SARS-CoV-2 variants pose risks causing uncertainty on the global economic recovery in 2021. As such the United Kingdom, previously expected to economically rebound in the first quarter of 2021, faces a GDP contraction of 4%, following lockdown 3.0 caused by the spread of the B.1.1.7 variant [342]. The IMF projects in a downside scenario, that the economic global growth would only recover to 1.6% in 2021 and 2.5% in 2022, if new COVID-19 cases remain high around the world and the vaccine rollout process is disrupted by logistical hurdles, new virus strains or public reluctance to vaccination [340]. Negative global growth in 2021 remains a possibility under a pessimistic scenario, in which financial stress is widespread [340].

As a result of the deep global economic recession, the World Bank estimates that in 2020, between 119 and 124 million people worldwide were pushed into extreme poverty, due to the COVID-19 pandemic [338]. The number of COVID-19 induced poor is estimated to increase between 143 and 163 million in 2021 [338]. The International Labour Organization (ILO) revealed that the COVID-19 pandemic led to an 8.8% loss in working hours in 2020, equivalent to 250 million full-time jobs [343]. According to the ILO estimates the working hours lost in 2020 were four times greater than during the 2009 financial crisis, translating into a global employment loss of 114 million jobs, increasing global unemployment by 33 million and reducing the global labour income by a total of USD3.7 trillion [343]. The ILO and IMF forecast under a baseline, pessimistic and optimistic scenarios that global working hours in 2021 would fall by 3.0%, 4.6% and 1.3% respectively, depending on the epidemiological situation [343]. Companies in impacted industries express their willingness to retain their employees if a time estimate is provided by the WHO, pertaining to when the outbreak will end. Currently, no confirmed time estimate exists [344].

Although the incomes of world states declined, governments are required to increase the budgetary allocations for their health sectors to combat the COVID-19 outbreak. The increase in government spending on healthcare is to facilitate hospitals, ICU units, isolation centers, equip medical facilities, procure the relevant drugs and testing kits, and accommodate citizens’ evacuated from abroad [345]. This shortage between government revenue and spending is an economic challenge that hinders the healthcare response to the pandemic’s outbreak. The virus outbreak in Italy reduced the state’s revenues from the tourism sector (accounts for 14% of GDP), creating a financial deficit. The financial deficit situation hindered the capacity of the Italian government to support its healthcare sector with sufficient resources and funds, resulting in the spread of the outbreak [346]. In addition, the action of global governments reprioritizing their budgets to support their health sectors with the necessary funds to combat the pandemic would result in a shortage in budgets allocated to other fundamental sectors, primarily education. The World Bank estimates the per capita education spending in all world countries shrunk by 5.7 percent in the second half of 2020 [347]. While there is no verified global estimate of the financial funds required to control the outbreak, WHO has requested USD 675 million to combat COVID-19 [348]. On June 26, 2020, the WHO announced that developing COVID-19 tests, treatments, and vaccines will require USD31.3 billion over the next one year. The requested financing will enable the delivery of 500 million tests and 245 million courses of treatment to low and middle-income countries over the next 12–18 months [349]. The WHO indicated in August 2020, that ensuring global access to SARS-CoV-2 vaccine will require over US$100 billion [350].

The decline in state revenue caused by the virus outbreak, limited resources, and medical infrastructure prevents developing states from independently financing the combat of the virus. Private financial inflows into the economies of developing countries has dropped in 2020 by a number of US$700 billion in comparison to the levels of 2019. This exceeds the impact of the Global Financial Crisis in 2008 by 60 percent, resulting in setbacks and reductions in global development of infrastructure creating a situation in which states would become more vulnerable to future crises particularly regarding health such as in the case of a future pandemic [351]. The pandemic has also caused a decline in African states export revenues, followed by depreciation in local currencies. A depreciation in exchange rates increases local inflation rates of foreign currency debt, which intensifies the situation of debt distress. Although governments are required to increase healthcare spending to combat the pandemic, African governments may be required to implement financial tightening measures to manage economic inflation [352]. Developing countries depend on donations from donor states, international organizations, and NGOs to attain the necessary funds to support their health care sectors in combating the COVID-19 outbreak. In this respect, the WHO introduced a crowdfund requesting the support of public and private donors for its COVID-19 response [353]. The World Bank also allocated USD14 billion to aid developing countries' COVID-19 response [354] and USD12 billion to finance their purchase and distribution of vaccines [354, 355]. However, the United States, which funded 15% of the WHO's 2018–2019 budget, announced on April 14, 2020, the suspension of its funding to the organisation. The US was due to pay USD58 million to the WHO in 2020 [356]. The US reversed its withdrawal decision, restoring its funding to the WHO in January 2021 [357].

To avoid the spread of the pandemic the WHO has urged developing states to implement containment procedures [358]. However, governments may resist implementing containment precautions, since countries that have imposed lock-downs and curfews have experienced economic repercussions, caused by the decline in volume of trade [359]. The United States for instance, whose economy contracted by 32.9% in the second quarter of 2020, did not implement containment measures in a timely fashion, due to its forecasted impacts on the national economy [360–362]. If implemented for the long term, the economic consequences of containment measures will be more intense on developing African states, considering their greater dependence on trade. The United Nations Economic Commission for Africa estimates that Africa’s GDP growth rate declines from 3.2% to 1.8% in 2020, pushing tens of millions in Sub-Saharan Africa into extreme poverty through 2021. The continent is expected to require at least USD100 billion as a fiscal stimulus to address the healthcare and economic needs associated with the pandemic [352]. Moving forward and in order to minimize the global recessionary gaps countries are advised to adopt collective international stimulus measures rather than independent actions. In this respect, the G20 and as part of an international coordinated action, announced the investment of USD11 trillion in the global economy to address financial losses caused by the virus outbreak [363, 364].

Worldwide governments encounter social and economic pressures to re-open economic activities, in order to account for increasing poverty rates and financial losses. The World Tourism Organization (UNWTO) revealed that the global tourism sector has lost a total of USD1.1 trillion in 2020 due to the COVID-19 outbreak [365]. Accordingly, several countries began in May 2020 to announce exit strategies for the COVID-19 pandemic, involving the relaxation of containment measures. Countries which re-open their economies however may be required to reimplement lockdown restrictions due to the resurgence of subsequent waves of COVID-19. The Authorities in France, UK, Ireland, Spain, Italy, Germany, Canada and China re-imposed restrictive lockdown measures during the end of 2020, due to cases surge and the spread of new virus strains [366]. Governments continue to examine the implementation of policies which would allow for the economy to reopen while avoiding a surge in COVID-19 new cases. Only returning employees aged 20–49, who encounter low fatality rates and the lowest risk of requiring hospitalization, back to the workplace is a proposed policy in countries where the healthcare system no longer has critical congestion. The risk of new waves of infections remains high nonetheless as a large fraction of the population is not immune to the virus. According to a report by the Imperial College COVID-19 Response Team (Ferguson et al. 2020), SARS-CoV-2 infection fatality rate in the age groups 20–29, 30–39, 40–49 is 0.03%, 0.08% and 0.15%, respectively. The corresponding probabilities of requiring hospitalization are 1.2%, 3.2% and 4.9% [367].

Other governments including Germany, Chile, and the USA proposed restarting the economy through issuing immunity passports, which certifies that an individual has been infected by SARS-CoV-2 and has developed antibodies to the virus. Following the rollout of COVID-19 vaccines, the International Air Transport Association (IATA) and several governments began issuing a digital vaccine passport for individuals who vaccinated against the virus, receiving all required doses [368]. Holders of vaccination passports could be allowed to resume economic and financial activities, while being exempt from physical restrictions. However, considerable scientific, practical, ethical and legal issues are posed by vaccination passports [367].

The global supply side of advanced pharmaceutical ingredients and pharmaceuticals was also disrupted by the virus outbreak. The closure of Chinese factories during the start of the pandemic resulted in a shortage of raw components used by international drug companies to develop essential vitamins and antibiotics. This shortage in the global supply of vital drugs may reflect negatively on the health of patients with diseases other than SARS-CoV-2. Additionally, measures taken by states to preserve their national stocks of vital medications during the outbreak resulted in an increased shortage of its global supply. For instance, the Indian government banned the pharmaceutical industry’s export of 26 drugs, antibiotics, and pharmaceutical ingredients, starting March 3, 2020; according to the India Brand Equity Foundation, one-fifth of the global exports of generic drugs in 2019 was supplied by India (worth USD19 billion of drug exports) [369]. On June 29, 2020, the United States’ government purchased 500,000 doses of the antiviral drug Remdesivir, accounting for 100% of Gilead Sciences’ global production of the drug in July, and 90% of the projected global stock in August and September 2020. Governments’ focus on marginalized advantages results in a global competition to secure access to SARS-CoV-2 drugs, which increases their global prices. As a result, a shortage in the global supply of Remdesivir is expected between July and September 2020 [370].

Additionally, the rollout of COVID-19 vaccines was complicated by global economic inequalities. Several high-income countries and due to their purchasing power secured enough doses to vaccinate its entire population multiple times in 2021, with 95% of world vaccines in January 2021 being administered in ten countries only [371]. The excess demand of vaccines by wealthy states, exceeding the market supply of vaccines by pharmaceutical companies, results in a shortage for developing and low-income countries. Estimates predict 90% of the population in 67 countries will be deprived from receiving the COVID-19 vaccine in 2021 [371]. The shortage of vaccine supply to world states was intensified by the EU decision to control the foreign exports of vaccines produced within the bloc [372]. According to the UN, vaccination policies lead to a rapid increase in the price of vaccines, resulting in countries such as South Africa paying 2.5 times higher price than the EU for the AstraZeneca/Oxford vaccine [372, 373].

The COVID-19 outbreak results in a negative financial outlook for global non-profit public healthcare sectors. The revenues of global health sectors are likely to decline in comparison to 2019, as a result of hospitals halting certain profitable medical services and elective surgical procedures in order to divert focus on cases linked to COVID-19. Hospitals in the US have an estimated loss of USD323.1 billion in 2020, and one million job loss in the American healthcare sector{Blumenthal, 2020 #202. Expenses will rise as a result of the demand to provide equipment to protect medical staff dealing with infected patients, as well as increased costs of employee upkeep. Under specific conditions, the financial losses of the health sector will be cushioned by government funding aimed at combating the COVID-19 pandemic [374]. However, government funding will not fully cover the financial losses that will be incurred by healthcare firms or the full cost of treating those infected [375]. The Spanish government, for instance, has taken measures to monopolize all private health industries and place it under the disposition of the national healthcare sector. The government did not set a time frame for providing financial reimbursement to private healthcare firms [283].

Nonetheless, the value of the financial budget that governments are required to allocate to support their health sectors in combating the pandemic outbreak differs from one country to another, depending on the current status of its health sector and the extent of its development and preparedness to cope with a pandemic. The fragile health systems of some developing countries are a result of their limited economic capacity, poor governance, and internal conflicts. This creates a situation in which the state’s health and economic resources are insufficient in combating the COVID-19 outbreak, while also continuing to combat the outbreak of other pre-existing epidemics in the country. Yemen for instance, while facing the novel SARS-CoV-2, must also deal with the Cholera epidemic and other communicable diseases (diphtheria, dengue fever and measles). Yemen has recorded 2 million suspected cases of Cholera as of January 2020 [376]. Yemen’s inflated economy, decreased government revenues, and its limited public healthcare system where only 50% of health facilities are functioning at full capacity, makes it incapable of combating multiple diseases at the same time [377]. The WHO requested a fund worth US$179 million from donor states to aid Yemen with the essential medical equipment to combat the COVID-19 outbreak [378]. Furthermore, countries' allocation of most of its health budgets towards combating COVID-19 has also led to a shortfall in budgets allocated for combating Malaria in sub-Saharan Africa where mass insecticide-treated net campaigns have been suspended [379].

Political contexts also factor into the economic capacity of countries to provide sufficient financial support to their healthcare sector. For example, Iran’s fragile healthcare system coupled with the economic lockdown it experiences due to politically-driven sanctions imposed by the UN and US, may have likely hindered the capacity of the Iranian government to support its health sector with the necessary funds for clinical, laboratory, and pharmaceutical equipment to efficiently combat the outbreak [326].

## Conclusion

The spread of COVID-19 has taken the world by surprise, and unlike most health crises of the recent decades, impacted the routine lives of all humans in all walks of life. Governments and national task forces continue to work with public health experts to contain the spread of the virus on a national level, through implementation of screening, quarantine, and community-based laws and protocols—despite the grave forecasted consequences on the economy. Multidisciplinary groups of scientists meanwhile are working on developing improved diagnostic tests, efficient health equipment, and effective vaccines. Healthcare workers, despite the personal risks associated with the global shortage in PPE, continue work on the frontlines to manage patients as per the published literature and official guidelines, focusing on sustaining respiratory function and preventing septic infections, coagulopathy, and cardiac morbidities—topics that clinical researchers continue to investigate in relation to COVID-19. The WHO continues to coordinate on an international level and disseminate evidence-based information and awareness. National efforts, essential workers, and communities at large, are cooperating with the aforementioned bodies, and each other, to see an end to this global health crisis. Although no end date can be predicted as of yet, once this pandemic is overcome through the collective global efforts, it will leave a significant residual impact behind, and many lessons to be learnt by all stakeholders involved—communities, governments, and health systems—As we move forward to deal with the next pandemic or global health crisis to threaten human existence, in what will hopefully be a more proactive, systematic, and efficient global response.

## Data Availability

Not applicable.
